# Digitalized suicide risk assessment in emergency psychiatry services

**DOI:** 10.3389/fpsyt.2026.1931317

**Published:** 2026-08-21

**Authors:** Lavinia Duica, Mihai Bran, Catalin Plesea-Condratovici, Pantelie Nicolcescu, Irina Antonescu, Nicolae Constantinescu

**Affiliations:** 1 Facultatea de Medicina, Universitatea Lucian Blaga din Sibiu, Sibiu, Romania; 2 Spitalul Clinic Coltea, Bucharest, Romania; 3 Facultatea de Medicina si Farmacie, Universitatea Dunarea de Jos din Galati, Galaţi, Romania; 4 Lucian Blaga University of Sibiu, Sibiu, Romania

**Keywords:** computational psychiatry, digitalized assessment, emergency psychiatric services, risk stratification algorithm, suicide risk

## Abstract

Emergency psychiatry services represent the primary point of contact for patients with acute suicidal risk; however, traditional risk assessment remains inconsistent and vulnerable to error.

**Objectives:**

This study aims to develop a comprehensive model for the digitalized assessment of suicide risk in emergency psychiatry services by integrating validated risk factors from the specialist literature into a clinical stratification algorithm. The study seeks to create a practical instrument that enables the rapid and standardized classification of patients into risk categories - low, moderate, high, and imminent - thereby supporting clinical decisions regarding the need for hospitalization and the intensity of therapeutic intervention.

**Methods:**

We developed a 30-factor stratification algorithm that integrates the following categories of factors: psychiatric disorders, weighted at 30%; psychological factors, weighted at 20%; personal history factors, weighted at 25%; socio-demographic factors, weighted at 10%; and acute contextual factors, weighted at 15%. The algorithm was developed on the basis of a synthesis of meta-analyses published between 2017 and 2025.

**Results:**

The conceptual scoring system ranges the level of risk factors (RF) from 0 to 100 points and uses provisional operational intervals for stratification: low risk, 0 ≤ RF ≤ 25; moderate risk, 25< RF ≤ 75; high risk, 75< RF ≤ 100; and imminent risk, defined by composite alarm conditions that override the numerical score. These intervals have not yet been clinically calibrated. The digitalized system also includes protective factors, which are considered separately in the recommendations section to assess the feasibility of the safety plan and continuity of care. This study reports the original development and formal specification of a conceptual, mathematical, and algorithmic model; it does not report clinical validation or patient-level predictive performance.

**Conclusions:**

The implementation of a digitalized system for suicide risk assessment in emergency psychiatry services may support clinicians in making clinical decisions regarding the psychiatric care of patients with acute suicidal risk in the short and medium term.

## 1 Introduction

Suicide represents one of the most serious public health challenges of the twenty-first century (1) and constitutes the second leading cause of death among young people aged 15 to 29 years worldwide (2). According to data from the World Health Organization, more than 700, 000 individuals die by suicide every year, corresponding to one death every 40 seconds. In the European context, suicide rates vary substantially across countries, with an average of approximately 11 cases per 100, 000 inhabitants per year, while Romania reports a suicide rate of 9.6 per 100, 000 inhabitants (3).

For every suicide death, it is estimated that there are approximately 20 suicide attempts (4), many of which require emergency medical care and have long-term consequences for the physical and mental health of survivors. The economic impact of suicide is also substantial, with direct and indirect costs estimated at billions of euros annually in developed healthcare systems. These costs include acute medical treatment, long-term care, loss of productivity, and the broader impact on families and communities.

Emergency psychiatry services play a crucial role in suicide prevention, frequently constituting the first, and sometimes the only, point of contact with the healthcare system for individuals experiencing an acute suicidal crisis. Studies indicate that approximately 40% of individuals who die by suicide had contact with emergency services during the month preceding death, while up to 80% had contact with healthcare services during the preceding year (5). However, many individuals who subsequently die by suicide present to medical services before death with a broad range of symptoms. In a study conducted in 2020, statistical evidence showed that, over a 10-year period, among a total of 1, 115 individuals who engaged in acts of self-harm, 28 - corresponding to 2.5% - died by suicide (6).

This reality underscores the critical importance of accurately assessing suicidal risk in emergency medical settings and highlights the need to develop more effective instruments for identifying and stratifying patients at risk. Emergency departments face major challenges in this regard, including high patient volumes, limited time available for assessment, variability in staff expertise in psychiatric evaluation, and the absence of standardized and validated protocols for suicide-risk triage (7).

The complexity of suicide risk factors represents another major challenge for emergency assessment. Suicidal behavior results from the complex interaction of biological, psychological, socio-demographic, and contextual factors, each of which may carry variable weight depending on the individual and the circumstances. The cognitive integration of these multiple factors and the estimation of cumulative risk constitute a difficult clinical task, one that is vulnerable to errors and cognitive biases. Studies have shown that clinicians tend to overestimate the importance of recently observed factors and underestimate more subtle or chronic risk factors, resulting in inconsistent risk assessments.

The present study aims to develop a comprehensive and practical algorithm for the digitalized assessment of suicide risk in emergency psychiatry services, specifically addressing the limitations identified in traditional approaches. The primary objective is to create an evidence-based risk stratification algorithm that integrates risk factors validated in the international literature into a standardized and readily interpretable scoring system. This algorithm will be designed for digital implementation, with an intuitive interface intended to guide medical staff through the assessment process and to serve as a decision-support instrument for generating clinical recommendations based on the identified level of risk.

The study will also contribute to the specialist literature by developing a protocol for the prospective validation of the algorithm in real-world clinical settings. The protocol will include specifications for sample-size calculation and data-collection procedures. This rigorous approach will enable the replication and independent validation of the system across diverse cultural and clinical contexts, thereby contributing to the development of a robust evidence base for the digitalized assessment of suicidal risk.

### 1.1 Systematic review of risk and protective factors

#### 1.1.1 Psychiatric factors

Recent meta-analyses have identified a hierarchy of suicide risk associated with different psychiatric disorders (8), with the highest risk estimates observed in affective disorders, psychotic disorders, substance use disorders, major neurocognitive disorder, and personality disorders.

Major depressive disorder represents the most prevalent psychiatric risk factor, being present in approximately 60% of individuals who die by suicide (9). The severity of the depressive episode correlates directly with suicidal risk, with suicide rates reaching up to 15% in severe depression (10). Specific features of the depressive episode that increase suicide risk include severe anhedonia, feelings of hopelessness, persistent insomnia, psychomotor agitation, and, in particular, the presence of suicidal ideation with a specific plan.

Bipolar disorder is associated with the highest suicide risk among all psychiatric disorders (11), with suicide mortality rates 15–20 times higher than those observed in the general population. Risk varies significantly according to the phase of the illness, being highest during mixed episodes, defined by the co-occurrence of manic and depressive symptoms, followed by severe depressive episodes and transitional phases between episodes.

Schizophrenia-spectrum disorders in which psychotic symptoms are present are associated with a suicide risk of approximately 5–10% (12), with the highest rates observed during the first years after illness onset and during periods of partial remission, when insight into the illness is preserved. Specific risk factors in schizophrenia include command hallucinations, particularly voices commanding suicide, persecutory or guilt-related delusions, comorbid depressive symptoms, and, especially, awareness of functional deterioration and prognosis.

Alcohol- and substance-related disorders are highly prevalent among individuals who attempt suicide. In clinical samples, alcohol use disorder consistently emerges as the second most frequent psychiatric diagnosis after depression among suicide attempters (13). According to a meta-analysis, the lifetime risk of suicide in alcohol dependence is 7% (14). The contribution of alcohol or other substances to suicidal behavior resides in behavioral disinhibition, poor impulse control, stress, depression, and the broader effects of social disorganization secondary to behavioral disturbances characteristic of addictive behavior (15).

With regard to personality disorders, statistical data indicate that the most frequent association with suicide, estimated at 8–10%, is observed in borderline personality disorder (16). In patients with borderline personality disorder, impaired regulation of affective states, such as depression, anxiety, and anger, together with self-destructive tendencies, further contributes to the clinical conditions that may facilitate suicide. The comorbidity of borderline personality disorder with affective disorders and/or substance abuse leads to a significant increase in the lethality of suicide attempts (17, 18).

Major neurocognitive disorder at onset or in its early stages is associated with an increased suicide risk (19) shortly after diagnosis, particularly among individuals with onset before the age of 65 and during the first 3–6 months following diagnosis. This risk is further potentiated by psychiatric comorbidities, especially depression, and is more prominent in the frontotemporal subtype, in which impulsivity and disinhibition may accelerate the transition from suicidal ideation to suicidal action.

#### 1.1.2 Transdiagnostic psychological factor

Within the vulnerability–stress paradigm, transdiagnostic psychological factors, such as hopelessness and impulsiveness, function as diatheses that facilitate the transition from baseline vulnerability to suicidality in the presence of acute precipitating stressors. Meta-analyses consistently show that hopelessness is significantly associated with suicidal ideation, suicide attempts, and even death by suicide, with small-to-moderate effect sizes, thereby confirming both its prognostic role and the limitations of its use as a single clinical predictor (20). With regard to impulsivity, recent syntheses indicate a robust, although overall modest, relationship with suicidality, more clearly evident in the transition from suicidal ideation to suicidal action. This supports the conceptual role of disinhibition in the volitional phase of suicidal behavior (21).

Hopelessness and impulsiveness are two independent factors significantly associated with suicidal behavior (22). Hopelessness has been independently and repeatedly associated with an increased risk of suicidal ideation, whereas impulsivity has been strongly associated with the risk of suicide attempts and death by suicide (23). While hopelessness is commonly present in major depressive disorder, the role of impulsivity in suicide is particularly linked to alcohol and substance use, as well as to Cluster B personality disorders (24).

#### 1.1.3 Life-history-related factors

Previous suicide attempts constitute a strong predictor of suicide (25). Among individuals who have engaged in suicide attempts, 12% subsequently died by suicide, with the risk being highest during the first two years following the attempt (26). Although non-suicidal self-injury (NSSI) is not accompanied by suicidal intent, it may nevertheless be considered a risk factor for suicide (27). A family history of suicide represents one of the strongest predictors of suicidal risk, increasing the risk two- to threefold, depending on the degree of kinship and the number of affected family members (28). Longitudinal studies have identified the period immediately following hospital discharge as one of maximal risk, with suicide rates during the first post-discharge week being 100 times higher than those observed in the general population (29).

Clinical studies have highlighted the contribution of childhood psychotraumatic experiences to the development of suicidal behavior. Emotional neglect, as well as physical or sexual abuse, is associated with increased impulsivity (30). All forms of childhood abuse, including physical abuse, sexual abuse, emotional abuse, and neglect, are strongly associated with suicidal ideation and suicide attempts both during adolescence and in adulthood, increasing suicide risk two- to fivefold compared with individuals without a history of trauma (31).

#### 1.1.4 Socio-demographic factors

Recent meta-analyses have clarified the magnitude of the associations between various socio-demographic factors and suicidal risk, thereby allowing a more precise weighting of these variables in predictive algorithms.

Gender represents a fundamental demographic factor in the epidemiology of suicide, characterized by a well-documented paradox deriving from the fact that men tend to use more violent and lethal methods, such as firearms and hanging, whereas women more frequently use medication overdose, which is associated with lower fatality rates. Thus, men exhibit rates of completed suicide three to four times higher than women, while women exhibit rates of suicide attempts two to four times higher than men (32). Age shows a complex relationship with suicidal risk, with distinct patterns across different age groups. Middle-aged adults, particularly those aged 45–64 years, have shown increasing suicide rates in recent decades, associated with economic stressors, chronic health problems, and difficult life transitions (33).

Socio-economic status represents a multidimensional risk factor that includes education, occupation, income, and residential status. The relationship between socio-economic status and suicide is complex and varies across cultural contexts. In general, unemployment increases suicide risk two- to threefold, with the highest risk occurring in the first months following job loss (34). Homeless individuals exhibit suicide rates up to 10 times higher than those of the general population, with contributing factors including comorbid psychiatric pathology, substance use, trauma, and limited access to healthcare services (35).

Marital status exerts a protective effect, with married individuals showing lower suicide rates than unmarried, divorced, or widowed individuals; this effect is more pronounced in men than in women (36).

Social support and isolation are environmental factors with a significant impact on suicide risk. Objective social isolation, such as living alone and lacking regular social contacts, as well as subjective isolation, including feelings of loneliness and perceived lack of support, independently increase suicide risk (37).

#### 1.1.5 Contextual factors

Recent stressful life events represent major determinants of suicidal behavior, with studies demonstrating a significant accumulation of stressors in the weeks and months preceding suicide attempts.

The death of a close person, divorce, or partner separation constitutes the category most strongly associated with acute suicidal risk (38, 39). Acute alcohol consumption substantially increases proximal risk: meta-analyses and recent studies indicate a dose–response association and an odds ratio of approximately 7 for suicide attempts following acute alcohol consumption; furthermore, 21–44% of individuals who die by suicide have a positive blood alcohol level (40). Severe interpersonal conflicts may precipitate impulsive suicidal behavior, particularly among adolescents and young adults (41). Legal problems, especially recent arrest or the prospect of incarceration, represent significant risk factors (42). A recent diagnosis of a severe or chronic illness, particularly medical conditions associated with chronic pain, progressive disability, or terminal prognosis, substantially increases suicidal risk (43, 44). Access to lethal means represents a critical environmental factor that influences not only the rate of suicide attempts, but especially their lethality (45).

#### 1.1.6 Protective factors

Protective factors include supportive relationships with family and friends, community involvement, active religious affiliation, and responsibilities toward others, particularly young children. A comprehensive assessment of the social context enables the identification of resources available for the safety plan, as well as the patient’s needs for social support.

Suicide risk assessment does not focus exclusively on the magnitude of risk factors, but also on protective factors, namely the internal and external resources that can be immediately mobilized to reduce risk. Clinical safety standards support this balance of factors, particularly because protective factors can be strengthened through interventions, such as consolidation of the support system (46). These factors, on the one hand, reduce the probability of transition from ideation to action and, on the other hand, provide operational reference points for intervention in the event of emerging suicide risk, for example, concrete resources that can be activated immediately within the safety plan.

Among the protective factors described in the literature, social connectedness is repeatedly identified as one of the most consistent and modifiable. Supportive relationships, including those with family and friends, community involvement, active religious belonging, and responsibilities toward others, especially young children, function as protective elements and can be directly translated into resources for the safety plan (47–49).

#### 1.1.7 Interactions and cumulative effects of risk factors and protective factors

Suicidal behavior rarely results from the influence of a single risk factor; rather, it emerges from the complex interaction and cumulative effects of multiple vulnerabilities and stressors. Contemporary models of suicidal risk, such as the stress–diathesis model (50) and the developmental trajectories model (51), conceptualize suicide as the outcome of the interaction between baseline vulnerability, represented by distal factors, and acute precipitating factors, represented by proximal factors (52). Prospective studies have identified “warning signs” that indicate the transition from suicidal ideation to suicide attempt (53), including a rapid increase in the intensity of suicidal ideation, the development of a specific plan, and behavioral preparations for suicide.

Epidemiological evidence indicates that multiple co-occurring factors can increase risk (54); however, risk depends not only on their number but also on their identity, domain, relative contribution, severity, temporal proximity, clinical context, and interactions. The proposed framework captures selected distinctions and priority rules, but its additive composite index does not yet estimate or validate all cross-domain interactions. For example, the protective effect of social support may be diminished in severe depression, while the impact of negative life events may be greater in an individual with a history of childhood trauma.

Understanding these interactions is crucial for the development of risk algorithms capable of reflecting the complexity of the suicidal phenomenon and enabling the identification of high-risk individuals even in the absence of severe individual risk factors.

## 2 Materials and methods

The heterogeneity of pathways to suicide requires a nuanced approach to risk assessment (55). Recent research has identified distinct subtypes of suicidal behavior, each characterized by specific patterns of risk factors and temporal trajectories. Planned suicide, characterized by prolonged ideation and detailed planning, is more frequently associated with chronic depression, hopelessness, and progressive functional deterioration (56). Impulsive suicide, characterized by a rapid transition from ideation to action, often within less than 10 minutes, is more frequently associated with personality disorders, acute substance use, and acute interpersonal stressors (57). These subtypes require different assessment and intervention strategies, with an emphasis on chronic factors and planning in the former case, and on the management of impulsivity and restriction of access to means in the latter.

### 2.1 Target population

The sample-size calculation takes into account the practical realities of longitudinal research in vulnerable populations. Initial recruitment targets 500–600 patients per center, with interim re-estimations.

The inclusion criteria were developed to capture the full spectrum of patients with suicidal risk presenting to mental health services. Eligible participants include patients aged over 18 years who present with active suicidal ideation, defined as specific thoughts of self-harm with intent to die; passive suicidal ideation, defined as a wish to die without current active suicidal thoughts, suicidal intent, a specific plan, or preparatory behavior; a suicide attempt within the previous seven days, irrespective of medical severity; preparatory behavior for suicide, such as writing a farewell letter or giving away possessions; or referral for emergency psychiatric assessment on the basis of suspected suicidal risk expressed by family members, friends, or professionals. The operational distinctions among ideation, intent, plan, and preparatory behavior are detailed in **Supplementary Appendix 1**, C-SSRS subsection.

The exclusion criteria are deliberately limited in order to maximize the external validity of the findings. Only patients with the following characteristics are excluded: severe cognitive incapacity preventing participation in the assessment, such as advanced dementia with an MMSE score ≤10, profound intellectual disability with an IQ ≤25, or severe delirium not reversible within 48 hours; insurmountable language barriers in the absence of a qualified translator; or explicit and persistent refusal to participate after receiving complete information about the study. Patients with acute intoxication are not excluded; rather, the assessment is postponed until they become capable of coherent participation, usually within 6–12 hours. Patients with active psychotic disorders are included as long as they are able to respond to the assessment questions, even if their responses are influenced by psychotic symptoms.

### 2.2 Assessment method

Traditional approaches to suicide-risk assessment, applied to all patients presenting to emergency services with a psychiatric condition, rely predominantly on unstructured clinical judgment or on the use of assessment scales, such as the Beck Scale for Suicide Ideation, the Suicide Intent Scale, or the Columbia Suicide Severity Rating Scale, including its C-SSRS Screen version. Both approaches have important limitations documented in the specialist literature. Although unstructured clinical judgment is valuable because it can integrate contextual nuances and subtle behavioral observations, it is substantially influenced by the clinician’s individual experience, cognitive biases, and situational factors.

Recent meta-analyses evaluating the performance of the most widely used suicide-risk scales in emergency settings indicate average values for sensitivity and specificity across most instruments, resulting in substantial rates of both false-positive and false-negative classifications. Positive predictive value remains particularly problematic, with fewer than 10% of patients identified as high risk subsequently making a suicide attempt during the follow-up period (58). This modest performance reflects both the complexity of the suicidal phenomenon and the difficulty of predicting a rare event within a heterogeneous population (59).

A digital assessment system may systematically guide the psychiatrist through a structured and standardized evaluation of multiple psychological and clinical domains, thereby facilitating the identification of risk factors through standardization of the assessment process (**Appendix 1, 2, 3**) and algorithmic integration of multiple risk factors.

Algorithms can integrate dozens of risk factors using empirically validated weights, thereby reducing the cognitive burden associated with mental integration and limiting the influence of cognitive biases. In addition, digital systems allow continuous updating of algorithms on the basis of new scientific evidence, systematic data collection for ongoing validation, and integration with electronic health record systems to enable rapid access to relevant medical history. The core algorithm uses a decision-tree structure augmented by weighted scoring, allowing both hierarchical stratification and gradual quantification of risk.

- Digitalized Assessment System.

The proposed system for the digitalized assessment of suicide risk was designed as a modular and scalable platform based on a three-layer architecture: the presentation layer, represented by the user interface; the logic layer, represented by the assessment algorithm and data-processing procedures; and the data layer, represented by secure data storage and integration into existing systems. The presentation layer provides a web-based interface with an intuitive design that guides the user through the assessment process by means of clear visual prompts and real-time validation of entered data. The logic layer implements the risk-stratification algorithm in a transparent and auditable manner, with each data entry and calculation documented to ensure complete traceability. The data layer ensures secure storage in accordance with GDPR standards, including end-to-end encryption and a complete audit trail of access events.

The digital workflow is intended to support, not replace, emergency clinical judgment. Reduced burden and rapid decision support are design objectives rather than demonstrated outcomes. The proposed two-stage workflow begins with the C-SSRS Screen Version and the minimum information required for immediate safety decisions and conditional-module triggering, followed by only the indicated D_1_–D_5_ modules and associated scales. Its feasibility in emergency clinical use has not yet been established.

- Risk-Stratification Algorithm.

The algorithm developed for risk stratification integrates 30 major risk factors organized into five main domains, each assigned a specific weight on the basis of evidence from recent meta-analyses. Within each domain, a scoring function is applied, followed by normalization for calculation of the normalized risk score.

Suicidal risk was operationalized through a score ranging from 0 to 100, structured across five domains, D_1_–D_5_, each domain having a predefined maximum weight, *PD_d_*, with the total summing to 100%: the domain of psychiatric disorders, D_1_, weighted at 30%; the domain of transdiagnostic psychological factors, D_2_, weighted at 20%; the domain of personal history of suicide attempts, D_3_, weighted at 25%; the domain of socio-demographic factors, D_4_, weighted at 10%; and the domain of acute contextual factors, D_5_, weighted at 15%.

The domain maxima were established *a priori* as literature-informed heuristic allocations, considering: 1) the strength and consistency of associations reported in the cited literature; 2) operational utility in emergency settings; and 3) the need to limit dominance by a single class of variables. D_1_ and D_3_ were assigned the largest shares, D_2_ and D_5_ intermediate shares, and D_4_ the smallest share. These numerical allocations were not estimated by model fitting, are not predictive probabilities, and remain provisional pending prospective calibration.

For each psychiatric disorder, an “epidemiological risk weight” was derived from the prevalence of suicide cases and the odds ratio associated with suicide in a given population. The proposed weighting scheme has not yet been empirically validated. Its performance will be evaluated in future studies.

Within each domain, the contribution of each factor was derived from data synthesized in the recent literature with respect to: 1) frequency in relevant samples, expressed as prevalence, p; 2) effect size, expressed as OR or RR with confidence intervals, synthesized from recent meta-analyses and population-based studies. For each factor, an “epidemiological risk weight” was derived from the prevalence of suicide cases and the odds ratio associated with suicide in a given population. The proposed scheme has not yet been empirically validated. Its performance will be evaluated in future studies (**Appendix 4**). For clarity, the literature-derived inputs are distinct from the literature-informed, *a priori* heuristic domain allocations and modeling coefficients; the current conceptual model is not clinically calibrated. The algorithmic specification is provided in **Supplementary Appendix 5**, Section 2–6, and performance will be evaluated prospectively through portal piloting, calibration, and iterative refinement.

Weighting and scoring parameters were developed, including *x*, the epidemiological weight; *w*, the relative weight; and *s*, the score. These parameters were obtained by normalizing individual contributions and projecting them onto the total weight assigned to each domain.

The normalized risk score for each risk factor was calculated as follows:

- First, the set of domains, indices, and constituent factors of domains *D*_1_-*D*_5_ was defined:
  *D* = {*D*_1_, *D*_2_, *D*_3_, *D*_4_, *D*_5_}, where *D* denotes the five domains of factors.
- The number of factors within each domain is denoted as follows: $D_{1}-n_{1},D_{2}-n_{2},D_{3}-n_{3},D_{4}-n_{4},D_{5}-n_{5}$, where *n_d_* represents the number of factors included in domain *D_d_*.
-
${\text{F}}_{\text{D}}=\overset{5}{\underset{\text{d}=1}{\cup }}{\text{F}}_{{\text{D}}_{\text{d}}}={\text{F}}_{\text{D}1}\cup {\text{F}}_{\text{D}2}\cup {\text{F}}_{\text{D}3}\cup {\text{F}}_{\text{D}4}\cup {\text{F}}_{\text{D}5}$ denotes the set of factors across all domains.
- The parameter *x_d_*, *_i_* is defined as the epidemiological weight of the factor located at position *i* within domain *D_d_*, where d∈{1,…,5}: where,
$$x_{d,i}=p_{d,i}\times OR_{d,i},$$
  *– p_d, i_* represents the prevalence of the risk factor located at position *i*, reported as a percentage (%), that is, the probability that this factor is present in the population under analysis; and,
  *– OR_d, i_* represents the odds ratio quantifying the increase in relative risk when the factor located at position *i* is present.
- *w_d,i_* denotes the relative, normalized weight of the factor located at position *i* within domain *D_d_*, that is, the weighting assigned to factor *i* in the total score such that all weights within the domain sum to 1: where,
$$w_{d,i}=\frac{x_{d,i}}{\sum_{k=1}^{n_{d}}x_{d,k}},$$
  *k* is an index ranging from 1 to the total number of factors included in domain *D_d_*.
- The parameter *s_d_*, *_i_* is defined as the partial score of the factor located at position *i*, namely its contribution to the total risk score before the application of presence indicators and domain-specific multipliers within domain *D_d_*: where,
$$s_{d,i}=PD_{d}\times w_{d,i},$$
  *PD_d_* represents the predefined weight assigned to domain *D_d_*.
- For each domain, the score may also include additional parameters:
${\mathrm{– Z}}_{i}\in \left\{0,1\right\}$ indicates whether the factor is present or absent within the respective domain α∈{1.00,0.60,0.30} denotes an attenuation coefficient used, where specified, to limit over-summation after factors have been ordered by the applicable domain rule. D_1_ uses the separate diagnostic-composition rule defined in Section 2.2.1, with the preserved outer wrappers *f_B_* = 0.70 and *f_C_* = 0.30 and the inner position coefficients *α_B_* = 1.00, *α_C_*_1_ = 0.60, and *α_C_*_2_ = 0.30.
  *– M*_sev_(*i*) denotes the severity multiplier for a given factor within a domain. Depending on intensity, according to predefined levels of severity, the factor may be classified as mild (1.00), moderate (1.10), or severe (1.20).
  *– M*_time_(*i*) denotes the temporal multiplier, assigned according to the temporal proximity of the event (1.00, 1.10, 1.20). This multiplier is applied in domains *D*_3_ and *D*_5_, although temporality is defined differently in these two domains.
  *– M*_state_(*i*) denotes the state multiplier. This multiplier is applied exclusively within domain *D*_1_ and refers to specific types of illness episodes that include clinical features potentially predisposing the patient to suicide (1.20).
  *– M*_sui_(*i*) denotes the provisional suicidogenic multiplier derived from the C-SSRS Screen Version: 1.00 for level 0; 1.10 for levels 1-2; 1.20 for level 3 or lifetime suicidal behavior; and 1.30 for levels 4–5 or a recent suicide attempt. Imminent risk is assessed separately through composite override conditions. NSSI in D_3_ is not assigned *M*_sui_; it is evaluated with *M*_sev_ and *M*_time_. Detailed operational mappings are provided in **Supplementary Appendix 5, Tables A4.4-A4.5**.
  – The imminent-risk indicator is calculated separately from RF and overrides the numerical score when any one of five sufficient routes is satisfied:
    1. C-SSRS level 5 and suicidal behavior within the last 3 months.
    2. D_1_ (psychiatric disorder), suicide plan or suicidal intent and suicidal behavior within the last 3 months.
    3. D_3_ (previous suicide attempt), suicide plan or suicidal intent and a suicide attempt within the last 12 months.
    4. D_5_ (contextual factor with M*_sev_*=1.2), suicide plan or suicidal intent and current suicidal behavior and acute alcohol intoxication or (BIS-11 > 80) or (BHS > 14).
    5. D_5_ (acute alcohol intoxication>0.15 g/dL), suicidal ideation with plan and/or intent or current/recent suicidal behavior and BIS-11≥80 or BHS>14.

In the case of imminent risk indicator, any satisfied route activates the crisis protocol regardless of RF; passive suicidal ideation alone does not activate this override. The fully parenthesized implementation is specified in **Supplementary Appendix 1, 2, 3, 5**, Section 13, **Table A4.6**, and Pseudocode A4.8.

#### 2.2.1 Psychiatric disorders domain (D_1_) (30% of the total score) assesses the presence and severity of acute psychiatric pathology

The psychiatric disorders domain (D_1_) quantifies the contribution of active psychiatric pathology at the time of assessment to the overall suicide-risk estimate. Within the emergency psychiatry service, a standardized suicide-risk screening procedure is applied to all patients presenting with mental health concerns, irrespective of whether suicidal ideation is initially verbalized, as well as to patients referred for emergency suicide-risk assessment at the request of family members, physicians from any medical specialty, or the police. The digital suicide-risk assessment is activated in patients who screen positive for suicide risk. The psychiatric interview conducted in the emergency department guides the psychiatrist toward a more comprehensive risk assessment within the psychiatric conditions identified during the clinical evaluation. These may include depressive disorders, bipolar disorder, alcohol or substance use disorders, Cluster B personality disorders, which may also be accompanied by adjustment disorder, psychotic disorders, trauma- and stressor-related disorders, obsessive-compulsive disorder, anxiety disorders, neurocognitive disorders, and other psychiatric conditions (**Table 1**).

**Table 1:** Psychiatric disorders domain (D_1_): state, severity, suicidogenic multiplier and indicators of imminent suicidal risk (abbreviations: severity multiplier (*M_sev_*), time multiplier (*M_time_*), state multiplier (*M_state_*)).

Psychiatric disorders (D_1_)	Scales	Evaluation criteria assessment
Depressive disorders (Major depressive disorder/Dysthymia)	PHQ-9	Severity multiplier (*M_sev_*) State multiplier (*M_state_*) Suicidogenic multiplier (*M_sui_*)
Bipolar disorder	YMRS	Severity multiplier (*M_sev_*) State multiplier (*M_state_*) Suicidogenic multiplier (*M_sui_*)
Alcohol/substance use disorder	AUS/DUS	Severity multiplier (abuse, dependence, severe dependence) (*M_sev_*) Suicidogenic multiplier (*M_sui_*_)_
Cluster B personality disorder (Borderline personality disorder, with or without adjustment disorder)	MSI-BPD	Severity multiplier (*M_sev_*) Suicidogenic multiplier (*M_sui_*_)_
Psychotic disorders	CRDPSS	Severity multiplier (*M_sev_*) State multiplier (*M_state_*) Suicidogenic multiplier (*M_sui_*_)_
Obsessive-compulsive disorder	DOCS-SF	Severity multiplier Suicidogenic multiplier (*M_sui_*_)_
Post-traumatic stress disorder	SPRINT (Short PTSD Rating Interview)	Severity multiplier (*M_sev_*) Suicidogenic multiplier (*M_sui_*_)_
Major neurocognitive disorder (onset or early stage only)	MoCa (The Montreal Cognitive Assessment)	Severity multiplier (*M_sev_*) Suicidogenic multiplier (*M_sui_*_)_

*The imminent-risk indicator: D_1_ (psychiatric disorder), suicide plan or suicidal intent and suicidal behavior within the last 3 months.

Longitudinal psychiatric history, including previous diagnoses, episodes, treatments, and hospitalizations, is collected to support clinical interpretation and auditability. It does not receive a separate numerical contribution in D_1_: only active psychiatric disorders are scored under the specified D_1_ rules, while the personal-history factors explicitly defined in D_3_ retain their existing contributions.

General Principles for Assessing the Psychiatric-Disorder and Suicidal-Behavior Score within D1.

- Clinical Assessment of Psychiatric Disorders and Suicidal Behavior within D_1_.

The assessment of mental status led to the establishment of a psychiatric diagnosis based on DSM-5-TR criteria (60), while the severity of psychiatric symptomatology was quantified by means of the previously mentioned scales (see **Appendix 2**), across three levels of severity: mild, moderate, and severe.

The assessment of psychiatric disorders that have been shown in the literature to be associated with suicide risk involves a series of standardized rating scales. These include, for example, depressive disorders, assessed using the Patient Health Questionnaire-9 (PHQ-9) (61); bipolar disorder, assessed using the Young Mania Rating Scale (YMRS) (62); alcohol/substance use disorder, assessed using the Alcohol/Drug Use Scale (AUS/DUS) (63); borderline personality disorder, assessed using the MacLean Screening Instrument for BPD (MSI-BPD) (64); psychotic disorders, assessed using the Clinician-Rated Dimensions of Psychosis Symptom Severity (CRDPSS) (65); post-traumatic stress disorder, assessed using the Short Post-Traumatic Stress Disorder Rating Interview (SPRINT) (66); obsessive-compulsive disorder, assessed using the Dimensional Obsessive-Compulsive Scale – Short Form (DOCS-SF) (67); and neurocognitive disorders, assessed using the Montreal Cognitive Assessment (MoCA) (68).

- Operationalization of Factors and Multipliers within Domain D_1_.

During the assessment of the patient’s mental status, a single psychiatric diagnosis may be identified; however, in most cases, there is a principal psychiatric diagnosis, namely the dominant psychiatric disorder at presentation, accompanied by secondary diagnoses.

- When several diagnoses are present, comprising a principal diagnosis and secondary diagnoses, the following rules should be observed:
- Clinical exclusivity for mood disorders: depressive disorders and bipolar disorder cannot be scored as comorbidities.
- Suicidal ideation and suicide attempts are not considered diagnoses.
- Substance-induced symptoms, such as substance-induced psychosis or mania, are scored under substance use disorder; psychosis or mania is not added separately as an autonomous diagnostic entity.
- Clinical specifiers, for example “with psychotic features” in depressive disorders or bipolar disorder, remain clinical characteristics of the principal diagnosis and are not scored as secondary diagnoses.

When a principal diagnosis, or base diagnosis (*B*), corresponding to the dominant active psychiatric disorder, is present together with multiple secondary diagnoses, or comorbidities (*C*), the latter are ranked according to their contribution to the patient’s psychopathological state. A maximum of two comorbidities may be scored. If more than two comorbidities are present, the first two are selected according to their psychopathological relevance.

The composite diagnostic score is calculated according to the following components:

- The diagnostic score (*s*), determined on the basis of prevalence and odds ratios reported in the statistical evidence available in the specialist literature.
- The diagnostic structure comprises either a single base diagnosis B or a base diagnosis B plus up to two comorbidities *C*_1_ and *C*_2_. The existing outer weighting factors remain *f_B_* = 0.70 for the base contribution and *f_C_* = 0.30 for the comorbidity block. Within those wrappers, the position coefficients are *α_B_* = 1.00, *α_C_*_1_ = 0.60, and *α_C_*_2_ = 0.30; any absent diagnostic term is 0. Comorbidities are ranked by psychopathological relevance.
- Specifiers of psychiatric symptom severity:
  – Severity multiplier (*M*_sev_), assigned according to the operational thresholds of the clinical rating scales, corresponding to mild, moderate, and severe levels.
  – State multiplier (*M*_state_), defined as the presence of a clinical episode containing specific clinical features, or combinations of clinical features, that increase suicide risk:
    1. In depressive disorders.
      - Severe episode with melancholic features.
      - Severe depressive episode with psychotic symptoms.
    2. In bipolar disorder:
      - Bipolar disorder, depressive episode with psychotic symptoms.
      - Bipolar disorder, manic episode with psychotic symptoms.
      - Bipolar disorder, manic episode with dysphoria.
    3. In psychotic disorders: Presence of command hallucinations with suicidal content:
      - Suicidogenic multiplier *M_sui_* is assessed using C-SSRS Screen Version: 1.00 for level 0; 1.10 for levels 1–2; 1.20 for level 3 or lifetime suicidal behavior; and 1.30 for levels 4–5 or a recent suicide attempt. Imminent risk is assessed separately. NSSI without suicidal intent remains a distinct D_3_ factor evaluated through *M_sev_* and *M_time_*. (**Supplementary Appendix 5, Tables A4.4-A4.5**).
      - Indicator of imminent suicidal risk: suicidal ideation with a suicide plan or suicidal intent, accompanied by suicidal behavior during the previous three months.

Operational Rules for Calculating the Psychiatric-Disorder Score within D_1_.

**Table 2** presents the prevalence values *p*_1, i_ and the corresponding odds ratios *OR*_1,i_ reported in the recent literature, which enable the calculation of *x*_1,_ *_i_*, representing the epidemiological weight; *w*_1,_ *_i_*, representing the relative weight; and *s*_1,_ *_i_*, representing the score assigned to each factor, in this case, each psychiatric disorder, within domain D_1_ (69–79).

**Table 2:** Psychiatric disorders domain (D_1_): derived weighting and scoring parameters for the factors included in D_1_ (*p*_1,_*_i_*, prevalence of factor *i* within domain D_1_; *OR*_1,_ *_i_*, odds ratio for factor *i* within domain D_1_; CI, confidence interval; *x*_1,_ *_i_*, epidemiological weight; *w*_1,_ *_i_*, relative weight; *s*_1,_ *_i_*, factor score).

Psychiatric disorders	*p*_1,_*_i_* (%)	OR_1, i_ (95% CI)	*x*_1, i_	*w*_1,_ *_i_*	*s*_1,_ *_i_*
Depressive disorders	36.7	11 (7.3-16.5)	4.0370	0.6677	20.030
Alcohol/substance use disorder	25	3.7 (2.8-5)	0.9250	0.1530	4.590
Psychotic disorders	4.9	7.8 (4.5-13.5)	0.3822	0.0632	1.896
Personality disorder	3.8	9 (5.6-14.4)	0.3420	0.0566	1.697
Bipolar disorder	7	4.6 (2.1-10.1)	0.3220	0.0533	1.598
Post-traumatic stress disorder	1	3.3 (2.73-4.04)	0.0330	0.0055	0.164
Obsessive-compulsive disorder	0.2	1.9 (1.9-4.2)	0.0038	0.0006	0.019
Major neurocognitive disorder (onset or early stage only)	0.1	1.28 (0.77-2.14)	0.0013	0.0002	0.006

- The set of factors included in domain D_1_ is defined as follows:
$$\begin{array}{c} {\text{F}}_{\text{D}1}=\{\text{depressive disorders, alcohol/substance use disorders,}\\ \text{psychosis, borderline personality disorders, bipolar disorder,}\\ \text{post-traumatic stress disorder, obsessive-compulsive disorder,}\\ \text{major neurocognitive disorder at onset or an early stage\}} \end{array}$$
- The derived weighting parameters for each factor within the domain are defined as follows:
$$x_{1,i}=p_{1,i}\times OR_{1,i}\;w_{1,i}=\frac{x_{1,i}}{\sum_{k=1}^{n_{1}}x_{1,k}}$$
$\sum_{k=1}^{n_{1}}x_{1,k}=6.0463$, where $i\in \left\{1,\ldots ,n_{1}\right\}$, and *n*_1_ denotes the number of factors included in domain D_1_.
- The score for domain D_1_, corresponding to psychiatric disorders, is obtained by projecting factor *i* onto the predefined domain weight *PD_i_*, where *PD_i_* = 30:
$$s_{1,i}=30\times w_{1,i}$$
- To calculate the general formula for the patient’s D_1_ domain score (*SD*_1_), the scoring rules specific to this domain are taken into account, namely:
  1. If multiple psychiatric disorders are present, they must be hierarchically ordered according to the clinical predominance of symptoms, distinguishing between the base diagnosis and comorbid diagnoses.
  2. The base diagnosis contributes 0.7 to the overall score, whereas comorbidities contribute 0.3. These contributions are expressed through the weighting factors *f_B_* and *f_C_*, respectively.
  3. No more than two comorbid diagnoses may be scored.
  4. Within the preserved outer wrappers *f_B_* and *f_C_*, the position coefficients are *α_B_* = 1.00 for the base diagnosis, *α_C_*_1_ = 0.60 for the first comorbidity, and *α_C_*_2_ = 0.30 for the second comorbidity; if a diagnostic term is absent, that term is 0.
$$\begin{array}{l} D_{1}=\left\{\text{Diagnosis}\left(\text{psychiatric disorder}\right)\right\}\\ \text{Diagnosis}=\text{Base Diagnosis}\left(B\right)+\text{Comorbid Diagnoses}\left(C\right)\\ \text{Diagnosis}=B+C_{1}+C_{2} \end{array}$$
- The general formula for the patient’s domain D_1_ score (*SD*1) is as follows:
$$\begin{array}{c} S_{D1}=\min(P_{D1},f_{B}\times \left(1.00\times s_{1,B}\times M_{sev}\left(B\right)\times M_{state}\left(B\right)\times M_{sui}\left(B\right)\right)\\ +f_{C}\times (0.60\times Z_{1}\times s_{1,C1}\times M_{sev}\left(C1\right)\times M_{sui}\left(C1\right)+0.30\times Z_{2}\times s_{1,C2}\times M_{sev}\left(C2\right)\times M_{sui}(C2))) \end{array}$$
  where:
  *• f_B_*: weighting factor for the principal, or base, diagnosis (*B*), with *f_B_* = 0.7.
  *• s*_1,_ *_B_*: score assigned to the principal diagnosis, namely the base diagnosis, within domain D_1_.
  *• M*_sev(_*_B_*_)_: clinical severity multiplier for the base diagnosis (*B*) within domain D1, with the following values: 1.00 for mild severity, 1.10 for moderate severity, and 1.20 for severe symptomatology.
  *• M*_state(_*_B_*_)_: state multiplier for the base diagnosis (*B*) within domain D_1_.
  *• M*_sui(_*_B_*_)_: suicidogenic multiplier for the base diagnosis (*B*) within domain D_1_.
  *• f_C_*: weighting factor for comorbidities (*C*), with *f_C_* = 0.3.
  *• α_C_*_1_: position coefficient for the first comorbidity, with *α_C_*_1_ = 0.60; *α_C_*_2_: position coefficient for the second comorbidity, with *α_C_*_2_ = 0.30. These coefficients operate inside the preserved *f_C_* = 0.30 block; if C_1_ or C_2_ is absent, its term is 0.
• Z∈{0,1}: the presence/absence indicator for comorbidity, where *Z* = 0 indicates the absence of comorbidity and *Z* = 1 indicates the presence of comorbidity.
  • $s_{1,C_{k}}$: score assigned to the comorbid diagnosis (*C_k_*) within domain D_1_.
$M_{\text{sev}\left(C_{k}\right)}$: clinical severity multiplier for the comorbid diagnosis (*C_k_*) within domain D_1_, with the following values: 1.00 for mild severity, 1.10 for moderate severity, and 1.20 for severe symptomatology.
${\mathrm{• M}}_{\text{sui}\left(C_{k}\right)}$: suicidogenic multiplier for the comorbid diagnosis (*C_k_*) within domain D_1_.
- A practical example involving the selection of D_1_ factors: a patient with a principal diagnosis, or base diagnosis (*B*), of major depressive disorder, specifically a severe depressive episode with psychotic features, and two comorbidities, namely severe borderline personality disorder and severe alcohol dependence, who presented with suicidal ideation accompanied by a specific plan.
$$S_{D1}=f_{B}\times \left(1.00\times s_{1,B}\times M_{state}\left(B\right)\times M_{sui}\left(B\right)\right)+f_{C}\times \left(0.60\times s_{1,C1}\times M_{sev}\left(C1\right)+0.30\times s_{1,C2}\times M_{sev}\left(C2\right)\right)$$
  SD1 = (0.70 * 1.00 * 20.030 * 1.20 * 1.30) + 0.30 * (0.60 * 4.590 * 1.20 + 0.30 * 1.697 * 1.20) = 21.872760 + 0.30 * (3.304800 + 0.610920) = 21.872760 + 1.174716 = 23.047476 ≈ 23.05.

#### 2.2.2 Psychological factors domain (D_2_) - 20% of the total score: hopelessness and impulsivity

Hopelessness and impulsivity are transdiagnostic factors of clinical relevance as diatheses and form a psychological-vulnerability domain that is operationally distinct from an active psychiatric diagnosis. This organization does not assume statistical independence between D_2_ and D_1_. These factors may facilitate the transition from baseline vulnerability to suicidality in the presence of acute precipitating stressors, and specified priority rules are applied where constructs overlap.

Longitudinal history of hopelessness and impulsivity is recorded as clinical context. D_2_ scores their current assessed state only, using the BHS and BIS-11 rules described above; no additional historical coefficient is introduced.

In the case of hopelessness, studies measuring its prevalence in general, non-clinical samples were considered, while samples consisting of psychiatric patients were avoided. The measurement of impulsivity was derived from a sample of suicide cases identified through medico-legal registries, including both clinical and non-clinical populations.

General Principles for Assessing the Psychological-Factor Score.

The assessment of hopelessness and impulsivity, as psychological factors relevant as diatheses for the occurrence of suicide attempts or suicide, is performed by means of standardized rating scales: the Beck Hopelessness Scale (BHS) (80) and the Barratt Impulsiveness Scale (BIS-11) (81). D_2_ may become the principal domain of psychological vulnerability, without being treated as statistically independent of D_1_, under the following conditions:

- If the patient presents with hopelessness-related cognitions and there is no active psychiatric diagnosis such as major depressive disorder, bipolar disorder, specifically a depressive or mixed episode, or dysthymia.
- With regard to impulsivity, psychological autopsy studies did not measure this construct in a non-clinical population; consequently, it was not possible to measure a dimension fully independent of the presence of a clinical diagnosis, such as bipolar disorder, alcohol/substance use disorder, or borderline personality disorder.
- The symptoms are transdiagnostic and are not constituent elements of clinical syndromes specific to a psychiatric disorder, such as depressive, depressive-anxious, dysphoric manic, mixed affective, or related syndromes.
- Individuals presenting with these transdiagnostic psychological factors within D_2_ may be assigned:
  1. a severity multiplier, according to the levels defined by the corresponding assessment scales; and.
  2. a suicidogenic multiplier, analogous to that applied to individuals included in the psychiatric-factors domain D_1_ (**Table 3**).

**Table 3:** Transdiagnostic psychological factors, assessment scales, and evaluation.

Transdiagnostic psychological factors	Scales	Evaluation criteria
Hopelessness	BHS	Severity multiplier (*M*_sev_) Suicidogenic multiplier (*M*_sui_)
Impulsivity	BIS-11	Severity multiplier (*M*_sev_) Suicidogenic multiplier (*M*_sui_)

Criteria BHS, Beck Hopelessness Scale; BIS-11, Barratt Impulsiveness Scale; *M*_sev_ = severity multiplier; *M*_sui_= suicidogenic multiplier.

Operational Rules for Calculating the Score of Transdiagnostic Psychological Factors $\left(F_{D_{2}}\right)$.

**Table 4** presents the scores assigned to the two psychological factors -hopelessness and impulsivity - within domain D_2_. These scores are calculated on the basis of prevalence estimates. *p*_2,_*_i_* and odds ratios *OR*_2,_*_i_*, synthesized from the recent literature (82–87), followed by the derivation of weighting parameters *w*_2,_*_i_*.

**Table 4:** Transdiagnostic psychological factors within domain D_2_: prevalence, odds ratios, epidemiological weights, relative weights, and factor scores.

Transdiagnostic psychological factors	*p*_2_*_, i_* (%)	OR_2,_*_i_* (95%CI)	*x*_2,_ *_i_*	*w*_2,_ *_i_*	*s*_2_ *_, i_*
Hopelessness	11	wOR 1.98 (1.46-2.69)	0.2178	0.2580	5.156
Impulsivity	30	2.09 (1.7-5.0)	0.6270	0.7428	14.844

Prevalence of factor *i* within domain D_2_; *OR*_2,_ *_i_*, odds ratio for factor *i* within domain D_2_; *wOR*, weighted odds ratio; CI, confidence interval; *x*_2,_ *_i_*, epidemiological weight; *w*_2,_ *_i_*, relative weight; *s*_2,_ *_i_*, factor score.

In the assessment of hopelessness, *wOR* is explicitly defined as the weighted odds ratio, that is, an odds ratio estimated using a weighted procedure, typically within a meta-analysis or another weighted analytic framework. The distinction is therefore not one of measurement units, but rather one of estimation method: *wOR* represents a weighted, aggregated estimate, whereas an *OR* may represent either an unweighted estimate or an estimate derived from a single study or model. Consequently, *wOR* may be used alongside *OR* without requiring conversion.

- The set of factors included in domain D_2_ is defined as follows:
$$F_{D_{2}}=\left\{\text{hopelessness, impulsivity}\right\}$$
- The derived weighting parameters for each factor located at position *i* are defined as follows:
$$x_{2,i}=p_{2,i}\times OR_{2,i}\;w_{2,i}=\frac{x_{2,i}}{\sum_{k=1}^{n_{2}}x_{2,k}}$$
$\sum_{k=1}^{n_{2}}x_{2,k}=0.8448$, where $i\in \left\{1,\ldots ,n_{2}\right\}$, and *n*_2_ denotes the number of factors included in domain D_2_.
- The score for the psychological-factors domain is obtained by projecting the factor located at position i onto the predefined domain score *PD*_2_, where *PD*_2_ = 20:
$$s_{2,i}=20\times w_{2,i}$$
- The general formula for the patient’s domain D_2_ score (*SD*_2_) is:
$$SD_{2}=\min\left(PD_{2},\sum_{k=1}^{n_{2}}\alpha_{k}\times Z_{2,k}\times s_{2,k}\times M_{sev}\left(k\right)\times M_{sui}\left(i\right)\right)$$
  where:
  *– k* denotes the position of the factor within domain D2, where $k\in \left\{1,\ldots ,n_{2}\right\}$.
  *– α_k_* denotes the coefficient indicating the ordered contribution of each factor within domain D2, applied in order to avoid over-summation, with: α={1.00, 0.60, 0.30, 0.20, 0.10}.
${\mathrm{– Z}}_{2,k}\in \left\{0,1\right\}$ denotes the presence/absence indicator for the factor located at position *k*.
  *– s*_2,_ *_k_* denotes the score assigned to the factor located at position *k*.
  *– M*_sev_(*k*) denotes the severity multiplier for the factors included in domain D_2_.
  *– M*_sui_(*i*) denotes the suicidogenic multiplier of factor ii, corresponding to either *n*_1_ or *n*_2_, within domain D_2_.
- A practical example, involving the selection of factors from D_2_: a patient presenting with severe hopelessness-related ideation (*BHS* = 24), suicidal ideation with a specific plan, and severe impulsiveness.
$$\begin{array}{l} SD_{2}=1.00\times Z_{2,1}\times s_{2,1}\times M_{\text{sev}\left(1\right)}\times M_{\text{sui}\left(1\right)}+0.60\times Z_{2,2}\times s_{2,2}\times M_{\text{sev}\left(2\right)}\\ SD_{2}=1.00\times 1\times 5.156\times 1.20\times 1.30+0.60\times 1\times 14.844\times 1.20=8.04+10.68=18.72 \end{array}$$

#### 2.2.3 Personal-history factors domain (D_3_) - 25% of the total score

The personal-history domain (D_3_) includes static risk factors that are nevertheless of particular relevance in predicting suicidal behavior, as they confer long-term vulnerability.

Previous suicide attempts and Non-suicidal self-injury (NSSI) are “static” predictors of recurrence; accordingly, they are assigned high internal weights, in line with longitudinal and meta-analytic evidence. Personal-history factors, including previous suicide attempts, self-harm, family history of suicide, childhood abuse, and previous psychiatric hospitalizations, were selected on the basis of recent meta-analyses and population-based studies.

A previous suicide attempt remains the most robust predictor of a future suicide attempt and of death by suicide (72). NSSI and childhood abuse were assigned substantial weights because of longitudinal evidence linking them to subsequent suicide attempts (70, 88). A family history of suicide or suicide attempts significantly increases risk because of genetic components, as well as psychological mechanisms related to behavioral modeling and exposure to trauma (89, 90).

The immediate post-discharge period is one of very high risk. Previous psychiatric hospitalizations indicate illness severity and recurrence and are associated with elevated suicide mortality during the first weeks and months following discharge (88, 91).

Principles for Assessing the Personal-History Factor Score.

The identification of these factors is considered in relation to the patient’s medical history, based on clinical history-taking, statements provided by relatives or caregivers, previous medical records, and other relevant documents, such as medico-legal certificates, police reports, and active protection orders.

In individuals whose medical history includes suicide attempts or acts of self-harm, studies have shown that these factors may vary in their predictive relevance for death by suicide depending on specific parameters. These include temporal multipliers, which capture the imminence of suicide risk according to the time elapsed since the incident, and intensity parameters, which reflect the severity of the injury or act and the degree of objective confirmation. Together, these parameters describe the circumstances under which the suicidal acts occurred (**Table 5**). The NSSI episode bands 1, 2–4, and ≥ 5 are ordinal, literature-informed heuristic thresholds specified provisionally; the ≥5 ceiling does not imply equal clinical risk for 5 and 50 episodes and will be assessed during prospective calibration.

**Table 5:** Personal history factors domain (D_2_): severity/time multipliers and indicators of imminent suicide risk [time multiplier (*M_time_*), severity multiplier (*M_sev_*)].

Personal-history factors domain (D_3_)	Evaluation criteria
Previous suicide attempts	Time multiplier (*M*_time_) 1.00 = 37–60 months; 1.10 = 13–36 months; 1.20 = ≤12 months. Severity multiplier (*M_sev_*) 1.00 = low lethality/superficial gestures, with no medical care required. 1.10 = moderate lethality, for example intoxication requiring medical treatment or cuts requiring sutures; two suicide attempts, or one clearly established attempt with suicidal intent. 1.20 = high lethality (hanging, firearm use, or jumping from a height) admission to an intensive care unit required; ≥3 suicide attempts. *Imminent risk: suicidal ideation combined with a suicide plan and/or suicidal intent, together with a suicide attempt within the previous 12 months
History of non-suicidal self-injury	Time multiplier (*M*_time_) 1.00 = 37–60 months; 1.10 = 13–36 months; 1.20 = ≤12 months. Severity multiplier (*M_sev_*) 1.00 = one episode involving superficial injuries, with no medical care required. 1.10 = two to four episodes, or one episode requiring minor medical care. 1.20 =≥5 episodes, or injuries requiring sutures and/or medical treatment (provisional ordinal threshold; prospective calibration required).
Family history of suicide	Severity multiplier (*M_sev_*) 1.00 = only distant relatives with suicide attempts or death by suicide. 1.10 = first-degree relative with a suicide attempt, or second-degree relative who died by suicide. 1.20 = first-degree relative who died by suicide, or ≥2 first- or second-degree relatives who died by suicide.
Childhood trauma severe physical, sexual, or emotional abuse	Severity multiplier (*M_sev_*) 1.00 = one type of moderate trauma. 1.10 = ≥2 types of moderate trauma, or repeated exposure to domestic violence. 1.20 = sexual abuse, ≥3 types of trauma, including physical abuse, emotional abuse and neglect with onset before the age of 12 years.
Previous psychiatric hospitalization	Severity multiplier (*M_sev_*) 1.00 = single hospitalization, with discharge occurring more than 12 months previously. 1.10 = multiple hospitalizations and/or clinically severe course, defined as ≥2 hospitalizations. 1.20 = recent discharge, defined as ≤3 months, or psychiatric hospitalization during the previous year.

Operational Rules for Calculating the Score of Transdiagnostic Psychological Factors $F_{D_{2}}$.

In **Table 6**, the prevalence estimates *p*_3,_*_i_* and odds ratios *OR*_3,_*_i_* assigned to personal-history factors are presented. These values were synthesized from the recent literature and enable the calculation of the derived weighting and scoring parameters within domain D_3_. As in the case of hopelessness reported in **Table 6**, the *wOR* value for previous psychiatric hospitalization may be used together with *OR* without requiring conversion.

**Table 6:** Personal-history factors domain (D_3_): derived weighting and scoring parameters for the factors included in D_3._ (*p*_3,_ *_i_*, prevalence of factor *i* within domain D_3_; *OR*_3,_ *_i_*, odds ratio for factor *i* within domain D_3_; CI, confidence interval; *x*_3,_ *_i_*, epidemiological weight; *w*_3,_ *_i_*, relative weight; *s*_3,_ *_i_*, factor score).

Personal-history factors (D_3_)	*_P_*_3,_*_i_* (%)	OR_3,_*_i_* (95%CI)	*x*_3_,i	*w*_3_ *,i*	*s*_3_,i
Previous suicide attempts	28.5	8.5(5.3-13.4)	2.4225	0.4083	10.208
History of non-suicidal self-injury	22.2	10.1(6.6-10.6)	2.2422	0.3779	9.448
Family history of suicide	11	3.7(2.3-5.7)	0.4070	0.0686	1.715
Childhood trauma severe physical, sexual, or emotional abuse	10	3.01 (1.19-7.09)	0.3010	0.0507	1.268
Previous psychiatric hospitalization	25	(wOR) 2.24 (1.69-2.97)	0.5600	0.0944	2.360

- The set of factors included in domain *D*_3_ is defined as follows:
$F_{D_{3}}$ = {previous suicide attempts, history of non-suicidal self-injury, family history of suicide, childhood trauma severe physical, sexual, or emotional abuse, previous psychiatric hospitalization}.
- The derived weighting parameters for each factor located at position *i* are defined as follows:
$$\begin{array}{c} x_{3,i}=p_{3,i}*OR_{3,i}\\ w_{3,i}=\frac{x_{3,i}}{\sum_{k=1}^{n_{3}}x_{3,k}} \end{array}$$
$\sum_{k=1}^{n_{3}}x_{3,k}\;=5.9327$, where $i\in \left\{1,\ldots ,n_{3}\right\}$ and denotes the number of factors included in domain D3.
- The score for the personal-history factors domain is obtained by projecting the factor located at position *i* onto the predefined domain score *PD*_3_, where *PD*_3_ = 25:
$$s_{3,i}=25*w_{3,i}$$
- The general formula for the patient’s domain D_3_ score (*SD*_3_) is as follows:
$$SD_{3}=\min\left(PD_{3},\sum_{k=1}^{n_{3}}\alpha_{k}*Z_{3,k}*s_{3,k}*M_{sev\left(k\right)}*M_{timp\left(k\right)}\right)$$
  where:
  *– k* denotes the position of the factor within domain D_3_, where: $k\in \left\{1,\ldots ,n_{3}\right\}$.
  *– α_k_* denotes the coefficient reflecting the ordered contribution of each personal-history factor, applied in order to avoid over-summation, with: α={1.00,0.60,0.30,0.20,0.10}
${\mathrm{– Z}}_{3,k}\in \left\{0,1\right\}$ denotes the presence/absence indicator for the factor located at position k.
  presence indicator for comorbidity, where *Z* = 0 indicates the absence of comorbidity and *Z* = 1 indicates the presence of comorbidity.
  *– s*_3,_ *_k_* denotes the score assigned to the factor located at position *k*.
  *– M*_sev_(*k*) denotes the clinical severity multiplier for the factors included in domain D_3_.
  *– M*_time_(*k*) denotes the time multiplier for the factors included in domain D_3_.
- A practical example, involving the selection of factors from D_3_: a patient with a high-lethality suicide attempt, by hanging, occurring six months prior to assessment; a history of five episodes of Non-suicidal self-injury (NSSI) during the previous year, which required medical intervention; a family history of suicide, namely a grandfather who died by suicide; a history of childhood sexual abuse; and a psychiatric hospitalization during the previous year.
$$SD_{3}=\left(\alpha_{1}*s_{3,1}*M_{sev\left(1\right)}*M_{timp\left(1\right)}+\alpha_{2}*s_{3,2}*M_{sev\left(2\right)}*M_{timp\left(2\right)}+\alpha_{3}*s_{3,3}*M_{sev\left(3\right)}+\alpha_{4}*s_{3,4}*M_{sev\left(4\right)}+\alpha_{5}*s_{3,5}*M_{sev\left(5\right)}\right)$$
  *SD*_3_ = 1 * (10.208 * 1.20 * 1.20) + 0.6 * (9.448 * 1.20 * 1.20) + 0.3 * (2.360 *1.2) + 0.2 * (1.716 * 1.20) + 0.1 * (1.268 * 1.20) = 14.78 + 8.05 + 0.84 + 0.41 + 0.15 = 24.2.

#### 2.2.4 Socio-demographic factors domain (D_4_) - 10% of the total score: integration of personal vulnerability factors within socio-demographic parameters

The socio-demographic factors included in the risk model were selected on the basis of meta-analyses and population-based studies published between 2017 and 2024 (92). The following factors were considered: male sex, risk age range, defined as 45–65 years, marital status, including being single, divorced, or widowed, occupational status, including lack of occupation or unemployment, low socio-economic status, low educational attainment, living environment, including rural residence or socio-economically deprived urban settings, and social isolation.

Principles for Assessing the Socio-Demographic Factor Score.

Given the high population prevalence of certain socio-demographic characteristics, namely male sex, age between 25 and 45 years, unmarried, divorced, or widowed marital status, rural or socio-economically deprived urban living environment, lack of occupation, low occupational and financial status, and educational attainment limited to primary or lower-secondary education, it was methodologically decided that only one variant of each socio-demographic factor would be considered. Accordingly, severity multipliers for the parameters under consideration were not included.

Operational Rules for Calculating the Socio-Demographic Factor Score.

**Table 7** presents the prevalence estimates *p*_4,_*_i_* and odds-ratio values *OR*_4,_*_i_*, synthesized from the recent literature, which enable the calculation of *x*_4,_ *_i_*, *w*_4,_ *_i_*, and *s*_4,_ *_i_*, corresponding respectively to the epidemiological weight, relative weight, and score of each factor, in this case each socio-demographic factor, within domain D_4_ (71, 72, 93–99).

**Table 7:** Socio-demographical factors domain (D_4_): severity/time multipliers and indicators of imminent suicide risk.

Socio-demographic factors (D_4_)	*p*_4,_*_i_* (%)	OR_4,_*_i_* (95% CI)	*x*_4,_ *_i_*	*w*_4,_ *_i_*	*s*_4,_ *_i_*
Gender (male)	69.6	1.75 (1.68-1.82)	1.2180	0.1385	1.385
Risk age (45-65 ani)	34	1.52 (1.45-1.59)	0.5168	0.0587	0.587
Marital status: unmarried, divorced, or widowed	51.9	1.92 (1.75-2.12)	0.9965	0.1133	1.133
Living environment: rural residence or socio-economically deprived urban setting	48	1.70 (1.49-1.95)	0.8160	0.0928	0.928
Occupational status: no occupation	51.6	3.8 (2.2.-6.5)	1.9608	0.2229	2.229
Low economic status	47	2.8 (2.2–3.7)	1.3160	0.1496	1.496
Low educational attainment	33.8	2.7 (1.7–4.2)	0.9126	0.1037	1.037
Social isolation	26.5	4 (1.5-5.4)	1.0600	0.1205	1.205

Prevalence of factor *i* within domain D_4_; *OR*_4,_ *_i_*, odds ratio for factor *i* within domain D_4_; *wOR*, weighted odds ratio; CI, confidence interval; *x*_4,_ *_i_*, epidemiological weight; *w*_4,_ *_i_*, relative weight; *s*_4,_ *_i_*, factor score.

- The set of factors included in domain D_4_ is defined as follows D_4_: $F_{D_{4}}$ = {gender, risk age, marital status, living environment, occupational status, economic status, educational status, social isolation}
- The derived weighting parameters for each factor located at position *i*:
$$\begin{array}{c} x_{4,i}=p_{4,i}*OR_{4,i}\\ w_{4,i}=\frac{x_{4,i}}{\sum_{k=1}^{n_{4}}x_{4,k}} \end{array}$$
$\sum_{k=1}^{n_{4}}x_{4,k}=8.7964$, unde $i\in \left\{1,\ldots ,n_{4}\right\}\;$ and n_4_ denotes the number of factors included in domain D_4_.
- The score for the socio-demographical factors domain is obtained by projecting the factor located at position *i* onto the predefined domain score *PD*_4_ (*PD*_4_ = 10),
$$s_{4,i}=10*w_{4,i}$$
- The general formula for the patient’s domain D_4_ (*SD*_4_):
$$SD_{4}=\min\left(PD_{4},\sum_{k=1}^{n_{4}}\alpha_{k}*Z_{4,k}*s_{4,k}\right)$$
  where:
  *– k* denotes the position of the factor within domain D_4_, where: $k\in \left\{1,\ldots ,n_{4}\right\}$.
  *– α_k_* denotes the coefficient reflecting the ranked contribution of each socio-demographic factor, applied in order to avoid over-summation, with: α={1.00,0.60,0.30,0.20,0.10} (five most relevant socio-demographical factors).
${\mathrm{– Z}}_{4,k}\in \left\{0,1\right\}$ denotes the presence/absence indicator for factor *k*.
  *– s*_4,_ *_k_* denotes the score assigned to the factor located at position *k*.
- A practical example involving the selection of factors from D_4_: a male patient, aged 45–65 years, unmarried, residing in a socio-economically deprived urban environment, unemployed, with low income, lower-secondary educational attainment, and living alone.
$$SD_{4}=\left(\alpha_{1}*Z_{4,1}*s_{4,1}\right)+\left(\alpha_{2}*Z_{4,2}*s_{4,2}\right)+\left(\alpha_{3}*Z_{4,3}*s_{4,3}\right)+\left(\alpha_{4}*Z_{4,4}*s_{4,4}\right)+\left(\alpha_{5}*Z_{4,5}*s_{4,5}\right)$$
  *SD*_4_ = (1 * 2.229) + (0.6 * 1.496) + (0.3 * 1.385) + (0.2 * 1.205)+(0.1*1.133)=2.229 + 0.897 + 0.415 + 0.241 + 0.113 = 3.895.

#### 2.2.5 Acute contextual factors domain (D_5_) -15% of the total score: proximal determinants of immediate risk that may facilitate the transition from suicidal ideation to suicidal action

The contribution of acute contextual factors to the occurrence of suicide must be understood within the framework of the vulnerability–stress model. Vulnerability factors may consist either of transdiagnostic psychological factors, such as hopelessness and impulsivity, as baseline vulnerabilities, or of psychiatric disorders, as active psychiatric risk factors. In the presence of an underlying vulnerability, acute precipitating stressors increase the risk of suicide.

Specific types of stressors are differentially weighted: major interpersonal loss, interpersonal conflict involving humiliation, victimization by physical violence, imminent legal problems, acute financial crisis, recent diagnosis of a severe medical condition, and acute alcohol intoxication (**Table 8**).

**Table 8:** Acute contextual factors domain (D_5_): severity/time multipliers and indicators of imminent suicide risk (abbreviations: *M*_time_ = time multiplier; *M*_sev_ = severity multiplier).

Acute factors contextual)	Evaluation criteria
Major interpersonal loss, death, separation	Time multiplier (*M*_time_) 1.00 = 31–90 days; 1.10 = 8–30 days; 1.20 = ≤7 days. Severity multiplier (*M*_sev_): 1.00 = report of an interpersonal loss, without clear specifications. 1.10 = anticipated death of a close relative, or separation with major emotional consequences. 1.20 = unexpected death of a primary attachment figure, or divorce with major consequences. *Imminent risk: suicidal ideation with a suicide plan and/or suicidal intent, or suicidal behavior, combined with major interpersonal loss and acute alcohol intoxication/BIS-11 > 80/BHS > 14
Severe interpersonal conflict (involving humiliation or rejection)	Time multiplier (*M*_time_) 1.00 = 31–90 days; • 1.10 = 8–30 days; • 1.20 = ≤7 days. Severity multiplier (*M*_sev_) 1.00 = report of interpersonal conflict, without clear specifications. 1.10 = no public humiliation, no threats, and no violence, but with marked emotional impact. 1.20 = public humiliation, threats, and/or police intervention. *Imminent risk: suicidal ideation with a suicide plan and/or suicidal intent, or suicidal behavior, combined with severe interpersonal conflict involving humiliation and acute alcohol intoxication > 0.15 g/dL or BIS-11 > 80/BHS > 14
Victim of physical violence	Time multiplier (*M*_time_) 1.00 = 31–90 days; • 1.10 = 8–30 day; • 1.20 = ≤7 days. Severity multiplier (*M*_sev_) 1.00 = report of physical violence, without clear specifications. 1.10 = physical assault with minor physical consequences, without mandatory medical care. 1.20 = physical assault, including sexual violence, requiring medical care. *Imminent risk: suicidal ideation with a suicide plan and/or suicidal intent, or suicidal behavior, combined with physical violence followed by substantial medical care and acute alcohol intoxication > 0.15 g/dL or BIS-11 > 80/BHS > 14
Imminent legal problems (arrest)	Time multiplier (*M*_time_) 1.00 = 31–90 days; • 1.10 = 8–30 day; • 1.20 = ≤7 days. Severity multiplier (*M*_sev_) 1.00 = report of legal problems, without clear specifications. 1.10 = restrictive measures with a near deadline, or a summons with a near deadline. 1.20 = arrest warrant issued, or conviction with immediate enforcement. *Imminent risk: suicidal ideation with a suicide plan and/or suicidal intent, or suicidal behavior, combined with imminent legal problems and acute alcohol intoxication > 0.15 g/dL or BIS-11 > 80/BHS > 14
Financial difficulties (bankruptcy, major debts)	Time multiplier (*M*_time_) 1.00 = 31–90 day; • 1.10 = 8–30 days; • 1.20 = ≤7 days. Severity multiplier (*M*_sev_) 1.00 = report of financial difficulties, without clear specifications. 1.10 = payment notices for overdue bank debts, registered bankruptcy proceedings, or dismissal notice. 1.20 = scheduled eviction or enforcement proceedings, bankruptcy, utility disconnection, job dismissal. *Imminent risk: suicidal ideation with a suicide plan and/or suicidal intent, or suicidal behavior, combined with major financial difficulties and acute alcohol intoxication > 0.15 g/dL or BIS-11 > 80/BHS > 14
Recent diagnosis of a severe medical condition	Time multiplier (*M*_time_) 1.00 = 31–90 days; 1.10 = 8–30 days; 1.20 = ≤7 days. Severity multiplier (*M*_sev_) 1.00 = stable or mild diagnosis, without major functional consequences. 1.10 = diagnosis of a severe disease, without meeting the criteria listed above. 1.20 = life-threatening or disabling diagnosis, or severe chronic pain. *Imminent risk: suicidal ideation with a suicide plan and/or suicidal intent, or suicidal behavior, combined with a recent diagnosis of a severe medical condition and acute alcohol intoxication > 0.15 g/dL or BIS-11 > 80/BHS > 14
Acute alcohol intoxication	Severity multiplier (*M*_time_) 1.00 = blood alcohol concentration< 0.08 g/*dL*; • 1.10 = blood alcohol concentration 0.08–0.15 g/dL; • 1.20 = blood alcohol concentration > 0.15 g/dL. *Imminent risk: suicidal ideation with a suicide plan and/or suicidal intent, or suicidal behavior, combined with acute alcohol intoxication with blood alcohol concentration > 0.15 g/dL or BIS-11 > 80/BHS > 14

Principles for assessing the acute contextual factor score.

Operational rules for calculating the acute contextual factors score.

**Table 9** presents the scores assigned to each factor *s*_5*,*i_, derived from the prevalence estimates *p*_5_, and odds-ratio values *OR*_5, i_ synthesized from the recent literature on acute contextual factors within domain D_5_ (40, 100–107).

**Table 9:** Acute contextual factors domain (D_5_): severity multipliers and indicators of imminent suicide risk (abbreviation: *p*_5,_ *_i_* prevalence of factor *i* within domain D_5_; *OR*_5,_ *_i_*, odds ratio for factor *i* within domain D_5_; CI, confidence interval; *x*_5,_ *_i_*, epidemiological weight; *w*_5,_ *_i_*, relative weight; *s*_5,_ *_i_*, factor score).

Acute contextual factors (D_5_)	*p*_5,_*_i_* (%)	OR_5,_*_i_* (95%CI)	*x*_5,_ *_i_*	*w*_5,_ *_i_*	*s*_5,_ *_i_*
Major interpersonal loss death, separation	29.5	1.2 (0.5-3.1)	0.3540	0.0865	1.297
Severe interpersonal conflict (involving humiliation or rejection)	21.1	5 (3.3-7.6)	1.0550	0.2580	3.870
Victim of physical violence	4	3.5 (2.4-5)	0.1400	0.0342	0.513
Imminent legal problems (arrest)	6.8	4.8 (2.4-9.4)	0.3264	0.0798	1.197
Financial difficulties (bankruptcy, major debts)	6	2.8 (2-4)	0.1680	0.0410	0.615
Recent diagnosis of a severe medical condition	19.1	2.9 (2.4-3.6)	0.5539	0.1354	2.031
Acute alcohol intoxication	21.4	6.97 (4.77-10.17)	1.4916	0.3647	5.470

- The set of factors included in domain D_5_:
$F_{D_{5}}$ = {major interpersonal loss death/separation, severe interpersonal conflict (involving humiliation or rejection, victim of physical violence, imminent legal problems (arrest, incarceration), financial difficulties (bankruptcy, major debts), recent diagnosis of a severe medical condition, acute alcohol intoxication}.
- Derived weighting parameters for each factor indexed by *i*:
$$\begin{array}{c} x_{5,i}=p_{5,i}*OR_{5,i}\\ w_{5,i}=\frac{x_{5,i}}{\sum_{k=1}^{n_{5}}x_{5,k}} \end{array}$$
$\sum_{k=1}^{n_{5}}x_{5,k}=4.0889$, where $i\in \left\{1,\ldots ,n_{5}\right\}$ and n_5_ denotes the number of factors included in domain D_5_.
- The score for the socio-demographical factors domain is obtained by projecting the factor located at position *i* onto the predefined domain score *PD*_5_ (*PD*_5_ = 15):
$$s_{5,i}=15*w_{5,i}$$
- The general formula for the patient’s domain D_5_ (*SD*_5_):
$$SD_{5}=\min\left(PD_{5},\sum_{k=1}^{n_{5}}\alpha_{k}*Z_{5,k}*s_{5,k}*M_{sev\left(k\right)}*M_{timp\left(k\right)}\;\right)$$
  where:
  *– k* - denotes the position of the factor within domain D_5_, where $k\in \left\{1,\ldots ,\;n_{5}\right\}$.
  *– α*_k_ - denotes the coefficient reflecting the ranked contribution of each acute contextual factor, applied in order to avoid over-summation, with: α={1.00, 0.60, 0.30, 0.20, 0.10} (five most relevant acute contextual factors).
${\mathrm{– Z}}_{5,k}\in \{0,1\}$ denotes the presence/absence indicator for factor *k*.
  *– s*_s,_ *_k_* denotes the score assigned to the factor located at position *k*.
  *– M*_sev_(*k*) denotes the severity multiplier for the factors included in domain D_5_.
  *– M*_time_(*k*) denotes the time multiplier for the factors included in domain D_5_.
- A practical example, involving the selection of factors from D_5_: a patient with acute alcohol intoxication, with a blood alcohol concentration of 1.2‰, who experienced the loss of his wife one week prior to assessment, had a conflict with his two children requiring police intervention, presented with major debts accompanied by threats of utility disconnection, and had stage III chronic heart failure.
$$SD_{5}=\left(\alpha_{1}*Z_{5,1}*s_{5,1}\right)+\left(\alpha_{2}*Z_{5,2}*s_{5,2}\right)+\left(\alpha_{3}*Z_{5,3}*s_{5,3}\right)+\left(\alpha_{4}*Z_{5,4}*s_{5,4}\right)+\left(\alpha_{5}*Z_{5,5}*s_{5,5}\right)$$
  *SD*_5_ = (1 * 5.47 · 1.2) + (0.6 * 3.87 * 1.2 * 1.2) + (0.3 * 2.03 * 1.2 * 1.2) + (0.2 * 1.29 * 1.2 * 1.2) + (0.1 * 1.19 * 1.2 * 1.2) = 6.56 + 3.34 + 0.87 + 0.37 + 0.17 = 11.31.

If the patient presents following a suicide attempt and at least one major acute contextual factor is identified within D_5_, the system requires the assessment of D_2_ factors, namely BHS and BIS-11, before generating the total score.

#### 2.2.6 Additional triggering and priority rules across domain D_5_–D_2_–D_1_:

These rules represent operational constraints of the digital system. They establish mandatory conditions for triggering specific scales and priority rules across domains, in order to prevent underestimation of risk and double counting of the same symptoms. If the patient presents following a suicide attempt and at least one major acute contextual factor is identified within D_5_, the system requires the assessment of D_2_ factors, namely BHS and BIS-11, before generating the total score. These are local operational priority and deduplication controls, not proof of statistical independence or of the absence of every possible cross-domain overlap. The applicable composite imminent-risk conditions are specified in **Supplementary Appendix 5**, Section 12–13; the contextual Boolean condition is fully parenthesized in Pseudocode A4.8.

The following notation is used:

-
$Z_{TS}\in \left\{0,1\right\}$: indicator of “presentation following a suicide attempt” at the time of assessment.
-
$Z_{5,i}\in \left\{0,1\right\}$: indicator of the presence of acute contextual factor *i* within D_5_.
- *τ_BHS_*: threshold corresponding to “at least mild” severity on the BHS.
- *τ_PHQ_*_–9_: threshold corresponding to “at least mild” severity on the PHQ-9.
- *τ_PHQ_*_–9_: threshold corresponding to “at least mild” severity on the PHQ-9.
- *τ_BIS_*_–11_: threshold corresponding to “at least mild” severity on the BIS-11.

- Rule 1: If $\left(Z_{TS}=1)\wedge (I_{D5}=1\right)$ then *SD*_2_ is mandatory, requiring administration of both the BHS and the BIS-11.
- Rule 2: If the BHS indicates at least mild severity: $BHS\geq \tau_{BHS}$, the system mandatorily triggers assessment of the patient using the PHQ-9, in order to verify the presence of an active depressive disorder. The following is defined:
$$Z_{TD}=\left[PH\mathrm{Q-}9\geq \tau_{PHQ-9}\right]$$
  1. In the presence of a depressive disorder (*Z_TD_* = 1), hopelessness is not scored separately within D_2_; instead, the corresponding score is taken from D1, for example: $s_{TD}=20.03$.
  2. In the absence of a depressive disorder (*Z_TD_* = 0), hopelessness is scored within D_2_, for example: $s_{H}=5.156$.
- Rule 3: If the BIS-11 indicates at least mild severity: $BI\mathrm{S-}11\geq \tau_{BIS-11}$ the impulsivity score is established within D_2_, for example: $s_{I}=14.844$ and the system does not automatically trigger administration of D_1_ scales for disorders that may include impulsivity, such as MSI-BPD or AUS/DUS. The impulsivity score from D_2_ is applied because it is higher than the scores assigned to psychiatric disorders in which impulsivity constitutes an important component, such as alcohol/substance use disorder, bipolar affective disorder, or borderline personality disorder.

## 3 Results

### 3.1 Total suicide-risk score

The total score is operationally defined as RF = D_1_ + D_2_ + D_3_ + D_4_ + D_5_, with each domain projected onto its predefined maximum of 30, 20, 25, 10, and 15 points, respectively, and RF bounded to [0, 100]. The sum is retained as a provisional composite index because it preserves this domain budget, remains transparent, and permits non-zero contributions when one domain is absent. A raw product was not adopted because it would collapse to zero whenever any domain score is zero and would require a new transformation, scale, and calibration. The additive form does not imply statistical independence and does not yet model all interactions; alternative aggregation and interaction specifications will be evaluated prospectively.

Through the distribution of domain weights, the introduction of modeling factors, and, not least, the use of general principles for assessing the score of each factor, the calculation of the total suicide-risk score allows psychological vulnerability and/or the acute contextual component to dominate the overall risk burden, even in the absence of a major psychopathological contribution at the time of assessment.

At the same time, the design maintains a clinical safety anchor through the introduction of “indicators of imminent suicidal risk”, which override the numerical score and directly trigger the crisis protocol, irrespective of the total score obtained. This limits the risk of underestimation in scenarios involving severe, acute, and clearly documented risk. Consequently, the total score should be used integratively, together with these flags and with the algorithmic recommendations generated according to risk levels, as a support for clinical decision-making rather than as a substitute for it.

This positioning is congruent with the NICE recommendation (2022) (108) to avoid the exclusive use of scales or risk stratification tools for prediction or discharge decisions. It is also supported by the literature on the predictive limitations of risk instruments, thereby justifying the interpretation of the total score as a composite index for clinical stratification and triage, rather than as a deterministic verdict regarding the suicidal behavior under assessment.

### 3.2 Contribution of protective factors to suicide risk

The integration of protective factors (*PF*) into a digital system designed to assess suicide risk should preferably not be implemented as a simple arithmetic “compensation” of risk, in the form: Risk = (D_1_+D_2_+D_3_+D_4_+D_5_) − PF. Studies indicate that the presence of protective factors may not counterbalance a significant acute risk determined by risk factors. For example, the protective effect of social support does not produce a linear reduction in suicide risk in severe depression; rather, protective factors may contribute to shaping the interactions between risk and protective factors in severe depression. For instance, suicide-risk assessment (109) requires the separate inventory of risk factors and protective factors. In the proposed model, protective factors are used in the recommendations section to assess the feasibility of the safety plan and the continuity of care.

Protective factors will be assessed using the Suicide Resilience Inventory-25 (SRI-25) (110) - a 25-item self-report questionnaire that measures factors protecting against suicidal thoughts and behaviors across three subscales: internal protective factors (coping ability), external protective factors (social and family support), and emotional stability (resilience). Within the proposed digital algorithm, the SRI-25 protective factor score can facilitate the mobilization of additional forms of support, thereby contributing to the safety plan (**Appendix 4**).

Much like SAFE-T (109) - which proceeds from the premise that protective factors do not counterbalance acute suicide risk but rather incorporates them into the recommendations section - the Digitalized Assessment System does not use protective factors as a mechanism for numerical compensation, but rather as a means of mitigating imminent suicide risk.

### 3.3 Integration of clinical data

The integration of the results generated by the Suicide Risk Stratification Algorithm into the clinical decision-making process is facilitated through the automatic generation of specific recommendations based on the identified level of risk.

Using the provisional operational intervals, low risk (0 ≤ RF ≤ 25) is linked to referral for an outpatient mental-health appointment; moderate risk (25< RF ≤ 75) is linked to medium- and long-term clinical monitoring, with admission considered when clinically indicated; and high risk (75< RF ≤ 100) is linked to psychiatric admission, with involuntary admission considered where appropriate. Any specified imminent-risk condition overrides RF and activates the crisis protocol, including close supervision and consideration of involuntary admission (**Table 10**). These recommendations support, but do not replace, clinical judgment.

**Table 10:** Suicide-risk levels and corresponding clinical recommendations.

Risk level	Recommendations
Low suicide risk (0 ≤ RF ≤ 25)	Referral for an appointment within the outpatient mental health service
Moderate suicide risk (25< RF ≤ 75)	Medium- and long-term clinical monitoring, with consideration of admission to a psychiatric unit where appropriate
High suicide risk (75< RF ≤ 100)	Admission to a psychiatric unit, with consideration of involuntary admission
Imminent risk presence of indicators of imminent suicidal risk	Activation of the crisis protocol, with consideration of involuntary admission where appropriate

Protective factors are integrated separately from the numerical score and are not used for arithmetic compensation of risk. Their role is to assess the feasibility of the safety plan and the continuity of care by identifying immediately mobilizable internal and external resources, including social support, family support, coping, resilience. A high PF score does not automatically reduce the risk category, but it increases the feasibility of outpatient management and implementation of the safety plan.

The system automatically generates a structured clinical report that includes the risk score, the risk category, the main determining factors, the relevant protective factors, and the corresponding recommendations. This report can be integrated into the medical record and serves as a support tool for clinical decision-making, ensuring traceability, consistency, and standardization in suicide-risk assessment within emergency services.

### 3.4 Worked synthetic end-to-end example.

The following fully synthetic case demonstrates deterministic application of the current formulas and is not a patient record or evidence of calibration, predictive validity, feasibility, or clinical utility. A 23-year-old woman presents with active suicidal ideation with a method, without a specific plan or intent, no suicidal behavior during the previous three months, and a blood alcohol concentration of 0.00 g/dL. The C-SSRS level is 3 and M_sui_ is 1.20. In D_1_, a moderate major depressive episode (PHQ-9 = 12) gives D1=min(30,0.70×20.030×1.10×1.00×1.20)=18.50772. In D_2_, hopelessness is not scored separately because active depression is already represented in D_1_; impulsivity (BIS-11 = 45) gives D2=min(20,1.00×0×5.156×1.20×1.20+0.60×1×14.844×1.00×1.20)=10.68768. In D_3_, one previous medication-overdose attempt 24 months earlier and one previous psychiatric hospitalization give D3=min(25,1.00×(10.208×1.10×1.10)+0.60×(2.360×1.00))=13.76768. In D_4_, unemployment, low economic status, social isolation, and unmarried status give D4=min(10,2.229+0.60×1.496+0.30×1.205+0.20×1.133)=3.71470. In D_5_, severe interpersonal conflict 15 days earlier and financial difficulties 45 days earlier give D5=min(15,1.00×(3.870×1.10×1.10)+0.60×(0.615×1.00×1.00))=5.05170. All five prespecified imminent-risk routes are false: the C-SSRS level is below 5, plan and intent are absent, no attempt occurred within 12 months, suicidal activation and alcohol are absent, and blood alcohol concentration is 0. The total is RF=18.50772+10.68768+13.76768+3.71470+5.05170=51.72948, reported as 51.73. Because 25< RF ≤ 75 and no imminent-risk route is activated, the final category is moderate. Protective factors are calculated and reported separately (PF = 8, moderate) and are not subtracted from RF.

### 3.5 Measurement of outcomes and impact

Screening and full-assessment durations have not yet been measured in clinical use. In the prospective pilot, Stage 1 will comprise the C-SSRS Screen Version and the minimum information required for immediate safety decisions and conditional-module triggering; Stage 2 will comprise the indicated D_1_–D_5_ modules, rating scales, priority and deduplication rules, imminent-risk flags, RF calculation, and separate PF assessment. Screening and full-assessment time will be measured separately from prespecified start and stop events. Clinician time, patient time, automated processing time, interruptions, incomplete assessments, completion rate, item and domain missingness, triggered-module frequency, manual clarification or override, and clinician-rated usability will be recorded as feasibility outcomes.

The planned portal pilot and subsequent prospective validation study will use a primary outcome defined as a suicide attempt or death by suicide during the 6-month follow-up period. This outcome will be ascertained through triangulation of direct reporting by the patient or family during follow-up contacts, electronic medical records for emergency consultations or hospital admissions, national mortality registries, and medico-legal reports in cases of suspected death.

The planned study will also assess secondary outcomes describing patients’ clinical trajectories. These will include re-presentation to emergency services for a suicidal crisis, including Nonsuicidal self-injury (NSSI); the rate and duration of psychiatric hospitalizations; adherence to outpatient treatment, measured by the proportion of attended appointments; and change in suicide-risk severity at a subsequent emergency presentation. These outcomes will be used to evaluate, rather than presume, the performance and clinical utility of the proposed system.

Prospective performance evaluation will assess rank discrimination with prespecified thresholds, sensitivity, specificity, positive predictive value, and negative predictive value. If RF is prospectively mapped to an estimated six-month probability, calibration-in-the-large, calibration slope, calibration plots, and the Brier score will also be evaluated. Inter-rater performance, prespecified subgroup performance, missingness, and workflow feasibility will be assessed. Endpoint definitions and adjudication, follow-up schedule, sample-size justification, missing-data handling, confidence-interval methods, exact inter-rater statistics, subgroup definitions and multiplicity handling, RF-to-probability mapping, model-updating rules, and the separation of calibration from external validation will be prespecified in the prospective protocol and statistical analysis plan.

Research in suicidology faces major challenges in distinguishing between different forms of self-injurious and suicidal behavior, particularly in differentiating suicide attempts from NSSI (111). The digitalized suicide-risk assessment system would enable the comprehensive evaluation of a substantial number of cases involving suicide risk, and the resulting database could contain clinically and epidemiologically relevant elements necessary for formulating judgments aimed at clarifying these clinical controversies.

## 4 Discussion

The algorithm proposed in this study aims to provide a clinically applicable stratification framework for patients at risk of suicide through the consistent and systematic application of evidence-based criteria. It is intended to support a standardized decision-making pathway, leading to the generation of an integrative clinical formulation. The proposed algorithm standardizes the elicitation and aggregation of clinical information, providing an explainable numerical output and operational thresholds for triage and safety planning.

The Suicide Assessment Five-step Evaluation and Triage framework, or SAFE-T (109), represents a protocol that integrates the assessment of risk and protective factors into a practical format for clinicians. The protocol guides the evaluator through five steps: identification of risk factors, identification of protective factors, assessment of suicidal ideation and behavior, determination of risk level, and documentation of the treatment plan. SAFE-T is not a scoring scale in the strict sense, but rather a model for organizing clinical information and guiding decision-making. This approach allows the integration of clinical judgment and adaptation to the patient’s specific context; however, it also introduces variability in assessment and makes standardization and inter-rater comparison more difficult. Implementation studies have shown that SAFE-T improves the completeness of assessment and documentation, although not necessarily the accuracy of risk prediction.

In contrast to SAFE-T, the proposed conceptual model specifies a weighted index and explicit provisional intervals that may support consistent documentation and map each risk category to recommended actions. A future digital interface is intended to improve traceability and documentation. Separating the 0–100 index from imminent-risk override rules provides a transparent implementation structure; it does not demonstrate predictive validity, calibration, or reduced inter-rater variability, which require prospective study.

OxSATS, the Oxford Suicide Assessment Tool for Self-harm, estimates the probability of death by suicide at 6 and 12 months after presentation for self-harm, based on approximately 11 social, demographic, and clinical factors. Its development and validation indicated good accuracy at 12 months while recommending further validation and linkage to interventions (112). OxMIS targets individuals with severe mental disorders through 17 predictors; external validations are emerging, although caution remains warranted regarding clinical utility (113). Whereas these tools provide medium-term probabilities, the present conceptual model is intended to provide real-time, literature-informed decision support for triage and safety planning; its predictive performance has not yet been established.

The proposed algorithm provides numerical standardization and rapid operational decisions within a digital workflow. It is intended as a decision-support instrument, not as a stand-alone decision system, for identifying imminent suicide risk, screening, triaging suicide-risk levels, and developing a personalized safety plan for patients at risk of suicide.

### 4.1 Limitations and anticipated challenges

Despite the substantial advantages of the digital system, one limitation of psychiatric assessment must also be emphasized: its accuracy may be affected by the provision of false or incomplete information.

An important limitation is that the current conceptual model has not been clinically validated or calibrated. The factor-level inputs are drawn from heterogeneous prevalence and effect estimates, whereas the domain allocations, attenuation coefficients, ordinal multipliers, additive aggregation, NSSI bands, and risk intervals are literature-informed heuristic choices specified a priori. RF is therefore a provisional stratification index, not an estimated probability of suicide. The planned next step is a pilot implementation of the portal in clinical practice, followed by prospective assessment of discrimination, calibration, inter-rater performance, and subgroup performance, iterative parameter refinement, and subsequent multicenter external validation. The present study reports no prospective performance results; all performance metrics listed above are planned evaluations.

Because completion-time, usability, and workflow-burden data are not yet available, operational feasibility in emergency settings remains to be established prospectively.

The NICE guidelines (113) discourage the use of scales or risk stratification tools, such as SAFE-T, for prediction or discharge decisions, and recommend that the algorithm be used as a support tool rather than as the sole arbiter of clinical decision-making. The design of the algorithm, which supports the suicide-risk score with clinical evidence and generates recommended rather than mandatory interventions, is consistent with this direction.

## 5 Conclusions

The present study proposes an original conceptual, mathematical, and algorithmic model for the digitalized assessment of suicide risk in mental health services. It integrates 30 literature-supported risk factors into five literature-informed domains: psychiatric, psychological, personal history, socio-demographic, and acute contextual. The original contribution lies in the structured combination and formalization of these factor classes, not in demonstrated clinical performance. Protective factors are assessed separately to inform the feasibility of the safety plan and continuity of care.

The conceptual model is accompanied by pseudocode in **Supplementary Appendix 5** to support reproducible implementation. The planned next stage is a pilot of the portal in clinical practice, followed by prospective calibration, iterative parameter refinement, and subsequent external validation. Research on clinical impact will also be needed before large-scale implementation. The model should therefore be interpreted as an original research output at the model-development stage. Clinical piloting, calibration, and external validation remain prospective. The digitalized suicide-risk stratification model remains a decision-support tool and not a substitute for clinical judgment.

## Data Availability

The original contributions presented in the study are included in the article/**Supplementary Material**. Further inquiries can be directed to the corresponding author.
