# Supplementary material for: Digitalized suicide risk assessment in emergency psychiatry services
Source: Front Psychiatry. 2026 Aug 21;17:1931317. doi: 10.3389/fpsyt.2026.1931317 (PMC13541670; doi:10.3389/fpsyt.2026.1931317)
Supplement: Supplementary file 1 [file SupplementaryFile1.docx]

**Supplementary files**

Appendix 1

| **Suicide multipliers** | **Evaluation criteria** |
| --- | --- |
| **Suicidal act severity-**  **Columbia Suicide Severity Rating Scale**  **Screen Version (C-SSRS)** | - Open access - Copyright protected |
|  |  |

Appendix 2

| Psychiatric disorders | Evaluation criteria |
| --- | --- |
| **Depressive disorders (Major Depressive Disorder/**  **Dysthymia) - Patient Health Questionnaire-9 (PHQ-9)** | - Open access - Permitted use |
| **Bipolar disorder - Manic/**  **Depressive/Mixed episode -**  **Young Mania Rating Scale (YMRS)** | - Open access - Copyright protected |
| Alcohol/Substance use disorder -  Alcohol Use Scale (AUS)/Drug Use Scale (DUS) | - Open access - Copyright protected |
| **Personality disorder from group B (Borderline personality disorder)**  **- Mac Lean Screening Instrument for BPD (**MSI-BPD**)** | - Copyright protected |
| **Psychotic disorders - Clinician-Rated Dimensions of Psychosis Symptom Severity (CRDPSS)** | - **Copyright protected** |
| **Obsessive-compulsive disorder -** Dimensional Obsessive-Compulsive Scale - Short Form  **(DOCS-SF)** | - Copyright protected |
| **Postraumatic stress disorder -**  Short Post-Traumatic Stress Disorder Rating Interview  **(SPRINT)** | - - - CC - Copyright protected |
| **Major neurocognitive disorder -**  **Montreal Cognitive Assesment (MoCA)** | - Open access - **Copyright protected** |

Appendix 3

| **Transdiagnostic psychological factors** | **Evaluation criteria** |
| --- | --- |
| **Hopelessness -**  **Beck Hopelessness Scale (BHS)** | - Copyright protected |
| **Impulsiveness - Barrat Impulsiveness** Scale (BIS-11) | - Open access - **Copyright protected** |

Appendix 4

| **Protective factors** | **Evaluation criteria** |
| --- | --- |
| **Protective factors -Suicide Resilience Inventory** | - Copyright protected |

Appendix 5

The digitized suicide-risk stratification algorithm: refactored specification, pseudocode, and non-regression controls

1. Purpose

Formalization of the algorithm into a specification implementable in a digital decision-support system for emergency psychiatry. The algorithm preserves the D_1_-D_5_ domains, the PD_d_ weights, the total RF score in the 0-100 range, imminent suicide-risk indicators, and the separate integration of protective factors PF.

The algorithm is a stratification and documentation tool, not a substitute for clinical judgment. If an imminent suicide-risk indicator is active, the numeric classification is overridden and the crisis protocol is activated, regardless of RF.

2. Canonical notation, equivalences, and global parameters

| Notation | Meaning | Implementation rule |
| --- | --- | --- |
| D={D_1_,D_2_,D_3_,D_4_,D_5_} | set of risk domains | according to the five-domain structure |
| FD_d_ | set of factors included in domain D_d_ | FD_1_...FD_5_ |
| n_d_ | number of factors in domain Dd | n_1_=8, n_2_=2, n_3_=5, n_4_=8, n_5_=7 |
| PD_d_ | maximum weight of domain Dd | PD_1_=30, PD_2_=20, PD_3_=25, PD_4_=10, PD_5_=15 |
| p_d,i_ | prevalence of factor i in Dd | if expressed as a percentage, divide by 100 when computing x_d,i_ |
| OR_d,i_ / wOR | odds ratio or weighted odds ratio | wOR is used directly, without conversion |
| x_d,i_ | epidemiological weight | xd_,i_=(p_d,i_/100)*OR_d,i_ for p expressed as a percentage |
| w_d,i_ | normalized relative weight | w_d,i_=x_d,i_/sum_k x_d,k_ |
| s_d,i_ | base score of the factor | s_d,i_=PDd*w_d,i_ |
| Z_d,i_ | presence/activation indicator | Z_d,i_ in {0,1}; equivalent to Z_i_, Z_k_, Z_2,k_ etc. in the text |
| αk | attenuation coefficient | α={1.00,0.60,0.30,0.20,0.10} |
| M_sev_ | severity multiplier | 1.00/1.10/1.20, according to the scale or domain criterion |
| M_time_ | temporal multiplier | used in D_3_ and D_5_; in D_3_ it does not apply to hospitalization |
| M_state_ | state multiplier | equivalent to Mst or Mstate; applies exclusively in D_1_ |
| M_sui_ | suicidogenic multiplier | derived from C-SSRS; imminent risk is treated separately |
| RF | Total Suicide-Risk Score | RF=SD_1_+SD_2_+SD_3_+SD_4_+SD_5_, limited to [0,100] |
| PF | protective-factor score | calculated separately; not subtracted from RF |

Table A4.1. Canonical notation and equivalences used in implementation.

| Domain | PDd | Content | Aggregation |
| --- | --- | --- | --- |
| D_1_ | 30 | Active psychiatric disorders | base diagnosis B + up to two comorbidities C_1_,C_2_ |
| D_2_ | 20 | Transdiagnostic psychological factors | hopelessness and impulsivity; fixed order H then I |
| D_3_ | 25 | Personal history | factors ordered by adjusted raw contribution |
| D_4_ | 10 | Socio-demographic | up to five factors, ordered by s_d,i_ |
| D_5_ | 15 | Acute context | up to five factors, ordered by adjusted raw contribution |

Table A4.2. Domains, weights, and main aggregation rules.

3. Base-score constants

The s_d,i_ constants can be recalculated from p_d,i_ and OR_d,i_ or loaded directly from the table. To reproduce the article values, p_d,i_ reported as a percentage is converted into a proportion before computing x_d,i_. For example, for depressive disorders: x_1,1_=(36.7/100)*11=4.037.

| D | i | Factor | p (%) | OR/wOR | x | w | s |
| --- | --- | --- | --- | --- | --- | --- | --- |
| D_1_ | 1 | Depressive disorders | 36.7 | 11 | 4.0370 | 0.6677 | 20.030 |
| D_1_ | 2 | Alcohol/substance use disorder | 25 | 3.7 | 0.9250 | 0.1530 | 4.590 |
| D_1_ | 3 | Psychotic disorders | 4.9 | 7.8 | 0.3822 | 0.0632 | 1.896 |
| D_1_ | 4 | Personality disorder / Cluster B-BPD | 3.8 | 9 | 0.3420 | 0.0566 | 1.697 |
| D_1_ | 5 | Bipolar disorder | 7 | 4.6 | 0.3220 | 0.0533 | 1.598 |
| D_1_ | 6 | Post-traumatic stress disorder | 1 | 3.3 | 0.0330 | 0.0055 | 0.164 |
| D_1_ | 7 | Obsessive-compulsive disorder | 0.2 | 1.9 | 0.0038 | 0.0006 | 0.019 |
| D_1_ | 8 | Major neurocognitive disorder | 0.1 | 1.28 | 0.0013 | 0.0002 | 0.006 |
| D_2_ | 1 | Hopelessness | 11 | wOR 1.98 | 0.2178 | 0.2580 | 5.156 |
| D_2_ | 2 | Impulsiveness | 30 | 2.09 | 0.6270 | 0.7428 | 14.844 |
| D_3_ | 1 | Previous suicide attempts | 28.5 | 8.5 | 2.4225 | 0.4083 | 10.208 |
| D_3_ | 2 | History of non-suicidal self-injury | 22.2 | 10.1 | 2.2422 | 0.3779 | 9.448 |
| D_3_ | 3 | Family history of suicide | 11 | 3.7 | 0.4070 | 0.0686 | 1.715 |
| D_3_ | 4 | Severe childhood trauma | 10 | 3.01 | 0.3010 | 0.0507 | 1.268 |
| D_3_ | 5 | Previous psychiatric hospitalization | 25 | wOR 2.24 | 0.5600 | 0.0944 | 2.360 |
| D_4_ | 1 | Gender: male | 69.6 | 1.75 | 1.2180 | 0.1385 | 1.385 |
| D_4_ | 2 | Risk age: AGE_RANGE_D_4_ | 34 | 1.52 | 0.5168 | 0.0587 | 0.587 |
| D_4_ | 3 | Unmarried/divorced/widowed | 51.9 | 1.92 | 0.9965 | 0.1133 | 1.133 |
| D_4_ | 4 | Rural/deprived urban residence | 48 | 1.70 | 0.8160 | 0.0928 | 0.928 |
| D_4_ | 5 | No occupation/unemployment | 51.6 | 3.8 | 1.9608 | 0.2229 | 2.229 |
| D_4_ | 6 | Low economic status | 47 | 2.8 | 1.3160 | 0.1496 | 1.496 |
| D_4_ | 7 | Low educational attainment | 33.8 | 2.7 | 0.9126 | 0.1037 | 1.037 |
| D_4_ | 8 | Social isolation | 26.5 | 4 | 1.0600 | 0.1205 | 1.205 |
| D5 | 1 | Major interpersonal loss | 29.5 | 1.2 | 0.3540 | 0.0865 | 1.297 |
| D_5_ | 2 | Severe interpersonal conflict | 21.1 | 5 | 1.0550 | 0.2580 | 3.870 |
| D_5_ | 3 | Victim of physical violence | 4 | 3.5 | 0.1400 | 0.0342 | 0.513 |
| D_5_ | 4 | Imminent legal problems | 6.8 | 4.8 | 0.3264 | 0.0798 | 1.197 |
| D_5_ | 5 | Financial difficulties | 6 | 2.8 | 0.1680 | 0.0410 | 0.615 |
| D_5_ | 6 | Recent severe medical diagnosis | 19.1 | 2.9 | 0.5539 | 0.1354 | 2.031 |
| D_5_ | 7 | Acute alcohol intoxication | 21.4 | 6.97 | 1.4916 | 0.3647 | 5.470 |

Table A4.3. The p_d,i_, OR_d,i_, x_d,i,_ w_d,i_, and s_d,i_ constants for the 30 factors.

4. Multipliers, thresholds, and mapping rules

Multipliers are applied only after the presence of the factor has been established. If the factor is absent, Z_d,i_=0 and its multipliers do not modify the score. Limit values that are not fully defined in the article are retained as protocol parameters and explicitly reported in the system output.

| Parameter | Values | Rule |
| --- | --- | --- |
| M_sev_ | 1.00 / 1.10 / 1.20 | mild / moderate / severe or the operational equivalent defined by the scale or domain criterion |
| M_time_ in D_3_ | 1.00 / 1.10 / 1.20 | 37-60 months / 13-36 months / ≤12 months; applicable to previous attempts and NSSI |
| M_time_ in D_5_ | 1.00 / 1.10 / 1.20 | 31-90 days / 8-30 days / ≤7 days; for acute alcohol intoxication, M_time_=1.00 |
| M_state_ | 1.00 / 1.20 | absent / episode with suicidogenic clinical features in D_1_ |
| M_sui_ | 1.00 / 1.10 / 1.20 / 1.30 | absent / C-SSRS level 1-2 / level 3 or lifetime suicidal behavior / level 4-5 or recent attempt |
| α | 1.00, 0.60, 0.30, 0.20, 0.10 | applied after ordering the relevant factors, to limit over-summing |

Table A4.4. Global multipliers and coefficients.

| Scale/criterion | Levels | Operational mapping |
| --- | --- | --- |
| C-SSRS | level 0; level 1-2; level 3 or lifetime suicidal behavior; level 4-5 or recent attempt | M_sui_=1.00; 1.10; 1.20; 1.30. Separate imminent risk: level 5 + suicidal behavior in the last 3 months. |
| PHQ-9 | 5-9; 10-14; ≥15 | M_sev_=1.00; 1.10; 1.20. τPHQ-9=5 for "at least mild". |
| YMRS | 13-19; 20-25; ≥26 | Ms_ev_=1.00; 1.10; 1.20. |
| AUS/DUS | 3=abuse; 4=dependence; 5=severe dependence/institutionalization | M_sev_=1.00; 1.10; 1.20. Levels 1-2 do not activate the D_1_ factor. |
| MSI-BPD | 1; 2; ≥3 | M_sev_=1.00; 1.10; 1.20. |
| CRDPSS | mild; moderate; severe | M_sev_=1.00; 1.10; 1.20. Equivocal symptoms do not activate the diagnosis unless there is an active clinical diagnosis. |
| DOCS-SF | mild; moderate; severe | M_sev_=1.00; 1.10; 1.20. |
| SPRINT | 0-10/11; 11-18; ≥19 | When thresholds overlap, the higher category is used. M_sev_=1.00; 1.10; 1.20. |
| MoCA | 18-25; 10-17; <10 | M_sev_=1.00; 1.10; 1.20, with reverse scale orientation: lower score = greater severity. |
| BHS | τBHS; moderate range; >14 | τBHS is a mandatory parameter for "at least mild"; BHS>14 activates the high severity used in the D_5_ rules. |
| BIS-11 | 30-51; 52-71; ≥72 | M_sev_=1.00; 1.10; 1.20. τBIS-11=30 for "at least mild". |
| D_5_ blood alcohol concentration | <0.08 g/dL; 0.08-0.15 g/dL; >0.15 g/dL | M_sev_=1.00; 1.10; 1.20. For the alcohol-specific imminent rule: BAC>0.15 g/dL. If the input is in ‰, convert it: g/dL=‰/10. |

Table A4.5. Mapping of scales and criteria to multipliers.

5. General data-control rules

•	An unknown factor is not automatically treated as absent. The system marks "missing/unknown" and blocks scoring if the factor can activate a mandatory scale, an imminent-risk indicator, or a category change

•	The RF score is not generated if the D_5_-D_2_-D_1_ rules require BHS, BIS-11, or PHQ-9 and the corresponding scale is missing

•	Contradictory data, for example negative C-SSRS but medical records indicating a recent attempt, are explicitly marked and require clinical confirmation

•	All RF, SD_d_, and PF values are reported with two decimals; rounding is not used before applying risk thresholds

6. General algorithm pseudocode

Pseudocode A4.1. General flow.

ALGORITHM SuicideRiskStratification(Patient P)

INPUT: clinical data; C-SSRS; diagnostic scales; D_3_-D_5_ data; PF data.

OUTPUT: R={SD_1_..SD_5_, RF, imminent_flags, risk_level, PF, recommendation}.

1. Validate mandatory data and assessment context.

2. Derive C-SSRS variables: SI_level, SI_plan_or_intent, suicidal_behavior_3m.

3. Apply D_5_-D_2_-D_1_ mandatory-trigger rules.

4. Evaluate imminent-risk flags: CSSRS, D_1_, D_3_, D_5__contextual, D_5__alcohol.

5. Compute SD_1_ = Compute_D_1_(P).

6. Compute SD_2_ = Compute_D_2_(P).

7. Compute SD_3_ = Compute_D_3_(P).

8. Compute SD_4_ = Compute_D_4_(P).

9. Compute SD_5_ = Compute_D_5_(P).

10. RF = min(100, max(0, SD_1_+SD_2_+SD_3_+SD_4_+SD_5_)).

11. PF = Compute_PF(P); do not subtract PF from RF.

12. IF any imminent flag is TRUE:

risk_level = "imminent risk"; recommendation = CrisisProtocol(PF)

ELSE:

risk_level = Classify_RF(RF); recommendation = Recommendation(risk_level, PF)

13. Return an auditable report with inputs, constants, intermediate scores, flags, PF, and action.

7. D_1_ domain: active psychiatric disorders

D_1_ quantifies the active psychiatric diagnosis. Clinical exclusivity rules apply: depressive disorder and bipolar disorder are not scored simultaneously as comorbid diagnoses; suicidal ideation and suicide attempts are not diagnoses; substance-induced psychosis or mania is scored under substance use disorder; clinical specifiers are not added as secondary diagnoses.

To make the general formula compatible with the D_1_ numeric example, a no-doubling rule is introduced: if the same clinical feature simultaneously activates M_state_(B) and M_sev_(B), M_sev_(B) is operationally set to 1.00 for the base diagnosis. This preserves the formula with M_sev_·M_state_·M_sui_, but avoids multiplying the same clinical characteristic twice.

Pseudocode A4.2. SD_1_ calculation.

FUNCTION Compute_D_1_(P):

PD_1_=30; fB=0.70; fC=0.30; alphaC=[1.00,0.60]

IF no active psychiatric diagnosis: RETURN 0

Apply diagnostic exclusivity and specifier rules.

B = principal/base diagnosis by clinical predominance.

C = up to two comorbid diagnoses by psychopathological relevance.

M_sev_B = SeverityMultiplier(B)

M_state_B = StateMultiplier(B)

IF SameClinicalFeatureActivates(M_sev_B, M_stare_B):

M_sev_B = 1.00

base = fB * s1[B] * M_sev_B * M_stare_B * M_sui_(B)

comorb = 0

FOR j in 1..min(2, len(C)):

comorb += alphaC[j] * Z(Cj) * s1[Cj] * M_sev_(Cj) * M_sui_(Cj)

RETURN min(PD_1_, base + fC * comorb)

The D_1_ imminent-risk indicator is: Imminent_D_1_ = (suicidal_plan OR suicidal_intent) AND suicidal_behavior_3m. The formula uses "plan and/or intent", not "plan and intent" as a cumulative requirement.

8. D_2_ domain: hopelessness and impulsivity

D_2_ is calculated in a fixed order: hopelessness at position 1, impulsivity at position 2. The factors are not sorted descending by s_d,i_, because the numeric example in the article applies α1 to hopelessness and α2 to impulsivity.

Pseudocode A4.3. SD_2_ calculation.

FUNCTION Compute_D_2_(P):

PD_2_=20; alphaH=1.00; alphaI=0.60

H = Z2,H * s2,H * M_sev_(H) * M_sui_(H)

I = Z2,I * s2,I * M_sev_(I) * M_sui_(I)

RETURN min(PD2, alphaH*H + alphaI*I)

// Regression anchor from article example:

// H: 5.156*1.20*1.30=8.04; I: 0.60*14.844*1.20=10.68; SD2=18.72.

9. D_3_ domain: personal history

D_3_ includes previous suicide attempts, NSSI, family history of suicide, severe childhood trauma, and previous psychiatric hospitalizations. M_time_ applies to previous attempts and NSSI. For family history, childhood trauma, and hospitalization, M_time_=1.00; recent discharge or hospitalization in the previous year is an Msev=1.20 criterion, not a separate temporal multiplier.

Pseudocode A4.4. SD_3_ calculation.

FUNCTION Compute_D_3_(P):

PD_3_=25; alpha=[1.00,0.60,0.30,0.20,0.10]

candidates=[]

FOR each factor k in FD_3_:

IF Z_3,k_=1:

IF k in {previous_suicide_attempts, NSSI}: mt=M_time_(k)

ELSE: mt=1.00

raw = s_3,k_ * M_sev(k)_ * mt

candidates.append((k, raw))

Sort candidates by raw descending.

total = sum(alpha[j]*candidates[j].raw for j=1..min(5,len(candidates)))

RETURN min(PD3, total)

The D_3_ imminent-risk indicator is: Imminent_D_3_ = (suicidal_plan OR suicidal_intent) AND previous_suicide_attempt_within_12_months.

10. D_4_ domain: socio-demographic factors

D_4_ does not use M_sev_, M_time_, M_state_, or M_sui_. Up to five present socio-demographic factors are selected, ordered by descending s_d,i_, and then α={1.00,0.60,0.30,0.20,0.10} is applied.

The age parameter is versioned as AGE_RANGE_D_4_. The article contains two formulations: 45-65 years in the domain description and example, and 25-45 years in the principles/table. The appendix implementation uses AGE_RANGE_D_4_=45-65 years by default to reproduce the domain description and example; if the final validated manuscript version confirms the table, the parameter is set to 25-45 years without changing the formula.

Pseudocode A4.5. SD_4_ calculation.

FUNCTION Compute_D_4_(P):

PD_4_=10; alpha=[1.00,0.60,0.30,0.20,0.10]

candidates=[]

FOR each factor k in FD_4_:

IF Z4,k=1: candidates.append((k, s_4_,k))

Sort candidates by s_4,k_ descending.

Keep at most five candidates.

total = sum(alpha[j]*candidates[j].score for j=1..min(5,len(candidates)))

RETURN min(PD_4_, total)

11. D_5_ domain: acute contextual factors

D_5_ includes proximal factors that may facilitate the transition from ideation to suicidal action. Present factors are ordered by the adjusted raw contribution raw5,k=s_5,k_*M_sev_(k)*M_time_(k), not only by s_5,k_. For acute alcohol intoxication, M_time_=1.00. Blood alcohol concentration must be stored internally in g/dL; if it is entered in ‰, the conversion is g/dL=‰/10.

Pseudocode A4.6. SD_5_ calculation.

FUNCTION Compute_D_5_(P):

PD_5_=15; alpha=[1.00,0.60,0.30,0.20,0.10]

candidates=[]

FOR each factor k in FD_5_:

IF Z_5,k_=1:

mt = 1.00 if k=acute_alcohol_intoxication else Mt_ime_(k)

raw = s_5,k_ * M_sev_(k) * mt

candidates.append((k, raw))

Sort candidates by raw descending.

Keep at most five candidates.

total = sum(alpha[j]*candidates[j].raw for j=1..min(5,len(candidates)))

RETURN min(PD_5_, total)

If the textual description of a case indicates financial difficulties, s_5,5_=0.615 is used. The s≈1.19 value belongs to the "imminent legal problems" factor (s_5,4_=1.197) and must not be transferred to financial difficulties. If the clinical example reports blood alcohol concentration in ‰, it is converted to g/dL before M_sev_ is established; for example 1.2‰=0.12 g/dL, meaning the 0.08-0.15 g/dL range, not >0.15 g/dL.

12. D_5_-D_2_-D_1_ trigger and priority rules

Pseudocode A4.7. D_5_-D_2_-D_1_ trigger rules.

FUNCTION Apply_D_5__D_2__D_1__Triggers(P):

ZTS = 1 if patient presents following a suicide attempt, else 0

ID5 = 1 if any acute contextual factor in D5 is present, else 0

IF ZTS=1 AND ID_5_=1:

require BHS and BIS-11 before RF generation

IF BHS >= tau_BHS:

require PHQ-9

ZTD = 1 if PHQ-9 >= tau_PHQ-9 AND active depressive disorder is confirmed, else 0

IF ZTD=1: Z2,H = 0	// hopelessness is captured through D1

ELSE: Z2,H = 1	// hopelessness is scored in D2

IF BIS-11 >= tau_BIS-11:

Z2,I = 1

// do not automatically trigger AUS/DUS, MSI-BPD, or bipolar scales only from BIS-11

13. Imminent suicide-risk rules

Imminent-risk indicators are calculated separately from RF and override the numeric score. Any of the conditions below is sufficient for classification as "imminent risk".

| Source | Operational condition | Action |
| --- | --- | --- |
| C-SSRS | C-SSRS level 5 plus suicidal behavior in the last 3 months | imminent=TRUE |
| D_1_ | suicidal ideation with plan and/or intent plus suicidal behavior in the last 3 months | imminent=TRUE |
| D_3_ | suicidal ideation with plan and/or intent plus suicide attempt in the last 12 months | imminent=TRUE |
| D_5_ contextual | suicidal ideation with plan and/or intent or current/recent suicidal behavior, major contextual factor (*M_sev_=1.2*), acute alcohol intoxication, and BIS-11>80 or BHS>14 | imminent=TRUE |
| D_5_ alcohol | suicidal ideation with plan and/or intent or current/recent suicidal behavior, BAC>0.15 g/dL and BIS-11≥80 or BHS>14 | imminent=TRUE |

Table A4.6. Override rules through imminent suicide risk.

Pseudocode A4.8. Contextual-imminence subroutine.

FUNCTION ContextualImminence(P):

suicidal_activation = SI_plan_or_intent OR suicidal_behavior_current_or_recent

high_D_2__state = (BIS11 > 80) OR (BHS > 14)

alcohol_present = Z5,acute_alcohol_intoxication=1

IF suicidal_activation AND (BAC > 0.15 g/dL) AND ((BIS-11 >= 80) OR (BHS > 14)):

RETURN TRUE

FOR k in {major_loss, severe_conflict, physical_violence, legal_problems,

financial_difficulties, severe_medical_condition}:

IF suicidal_activation AND MajorContextualFactor(k) AND alcohol_present AND high_D_2__state:

RETURN TRUE

RETURN FALSE

14. Total score, classification, and recommendations

Pseudocode A4.9. RF score classification.

FUNCTION Classify_RF(RF):

IF RF < 25: RETURN "low risk"

ELSE IF RF <=75: RETURN "moderate risk"	// fills the article gap at RF=25

ELSE: RETURN "high risk"

FUNCTION Recommendation(risk_level, PF):

IF risk_level="low risk":

return "outpatient mental-health appointment"

IF risk_level="moderate risk":

return "medium-/long-term monitoring; consider admission if clinically indicated"

IF risk_level="high risk":

return "psychiatric admission; consider involuntary admission where appropriate"

IF risk_level="imminent risk":

return "activate crisis protocol; close supervision; consider involuntary admission"

| Level | Deterministic criterion | Recommendation |
| --- | --- | --- |
| Low risk | RF <25 and no imminent indicator | outpatient mental-health appointment |
| Moderate risk | 25≤RF≤75 and no imminent indicator | medium-/long-term clinical monitoring; admission if clinically indicated |
| High risk | RF>75 and no imminent indicator | psychiatric admission; consideration of involuntary admission |
| Imminent risk | any imminent indicator | crisis protocol, close supervision, consideration of involuntary admission |

Table A4.7. Risk levels and recommendations.

15. Protective factors

PF is calculated separately and does not produce arithmetic compensation of RF. Its role is to estimate the feasibility of the safety plan and continuity of care over the next 24-72 hours.

Pseudocode A4.10. PF calculation.

FUNCTION Compute_PF(P):

PF=0

FOR each protective factor j in {coping, reasons_for_living, family_friend_support,

treatment_support, community_connectedness,

community_mental_health_network}:

Rj = robustness: 0 absent, 1 fragile, 2 robust

Aj = mobilization: 0 not mobilizable in 24-72 h, 1 mobilizable in 24-72 h

PFj = Rj * Aj

PF += PFj

IF PF <=4: PF_level="low"

ELSE IF PF <=8: PF_level="moderate"

ELSE: PF_level="high"

RETURN {PF, PF_level, PFj}

16. Structure of the automatically generated clinical report

| Section | Minimum content |
| --- | --- |
| Evaluation data | date/time, evaluator, context of presentation, information sources |
| Scores | SD_1_, SD_2_, SD_3_, SD_4_, SD_5_, RF, numeric level, thresholds used |
| Main factors | ordered factors, sd,i, multipliers, αk, and adjusted contribution |
| Imminent risk | C-SSRS, D_1_, D_3_, D_5_ contextual, D5 alcohol flags and the activated condition |
| Triggered rules | D_5_-D_2_-D_1_, mandatory scales, missing or contradictory data |
| Protective factors | PF, PF_level, mobilizable resources and absent resources |
| Recommendation | suggested action, need for admission, considerations regarding the safety plan |

Table A4.8. Minimum structure of the auditable report.

17. Regression controls

The controls below must be run after any change to the algorithm, the constants, or the threshold mapping. They do not clinically validate the model; they only verify that the implementation remains faithful to the specification and does not reintroduce the flagged errors.

| ID | Control | Deterministic anchor | PASS criterion |
| --- | --- | --- | --- |
| R01 | Number of factors | n_1_+n_2_+n_3_+n_4_+n_5_=30 | PASS if the total is 30 |
| R02 | Domain weights | PD_1_+PD_2_+PD_3_+PD_4_+PD_5_=100 | PASS if the sum is 100 |
| R03 | s_d,i_ sums | sum_D_1_=30.000; sum_D_2_=20.000; sum_D_3_≈24.999; sum_D_4_=10.000; sum_D_5_≈14.993 | PASS with tolerance 0.01 |
| R04 | Formula x | x=(p/100)*OR for percentage p | PASS if D_1_ depression x=4.037 |
| R05 | D_2_ without sorting | H receives α1 and I receives α2 | PASS if the D_2_ example gives 18.72 |
| R06 | D_1_ no-doubling | M_sev_(B) does not duplicate Ms_tate(_B) for the same trait | PASS if the D_1_ example reproduces 23.88 |
| R07 | D_3_ hospitalization | recent discharge is M_sev_=1.20, M_time_=1.00 | PASS if M_time_ does not appear for hospitalization |
| R08 | D_5_ ordering | sorting by raw=s*M_sev_*M_time_ | PASS if raw, not s, determines the order |
| R09 | D_5_ financial/legal | financial=s5,5=0.615; legal=s5,4=1.197 | PASS if 1.197 is not transferred to financial |
| R10 | D_1_/D_3_ imminence | plan OR intent, not plan AND intent | PASS if the operator is OR |
| R11 | D_5_ alcohol imminence | BAC>0.15 and BIS-11≥80 or BHS>14 | PASS if the rule is separate from contextual |
| R12 | PF separate | RF is not modified by PF | PASS if RF_before_PF=RF_after_PF |
| R13 | RF=25 | the undefined point interval is assigned to moderate | PASS if Classify_RF(25)=moderate |
| R14 | AGE_RANGE_D4 | versioned parameter, default 45-65 | PASS if the value is reported explicitly |
| R15 | Blood alcohol unit | BAC is evaluated in g/dL; if the input is in ‰, g/dL=‰/10 | PASS if 1.2‰ is interpreted as 0.12 g/dL |

Table A4.9. Minimum set of regression controls.

Factor 1: Internal Protective

1. I like many things about myself .80 .00 .00

2. Most of the time I am happy .77 .00 .00

5. I like myself .85 .00 .00

9. Most of my goals are reasonable .51 .00 .00

11. I am satisfied .79 .00 .00

13. I am proud of my good qualities .78 .00 .00

19. I feel emotionally strong .68 .00 .00

20. I am happy regardless of problems .80 .00 .00

25. I feel cheerful .88 .00 .00

Factor 2: Emotional Stability

4. I can deal with rejection .00 .74 .00

8. I can resist thoughts of suicide .00 .80 .00

10. I can resist suicidal urges when sad .00 .79 .00

12. I can resist suicidal thoughts when in a

difficult situation

.00 .79 .00

14. I can control suicidal urges when

criticized

.00 .79 .00

18. I can handle suicidal thoughts

when lonely

.00 .74 .00

23. I can resist suicidal thoughts when

humiliated

.00 .73 .00

24. I can resist suicidal thoughts when feeling

hopeless

.00 .81 .00

Factor 3: External Protective

3. People close would listen if I were suicidal .00 .00 .53

6. I could openly discuss suicidal thoughts .00 .00 .52

7. I can find someone for support .00 .00 .64

15. I can ask for support if suicidal .00 .00 .79

16. I can ask for support even if people are

angry with me

.00 .00 .60

17. I can find help if I am suicidal .00 .00 .83

21. If I am in trouble I can find help .00 .00 .79

22. Close friends or family would help me if

suicidal
